# Design, Synthesis and Biological Evaluation of Novel Phenylsulfonylurea Derivatives as PI3K/mTOR Dual Inhibitors

**DOI:** 10.3390/molecules23071553

**Published:** 2018-06-27

**Authors:** Bingbing Zhao, Fei Lei, Caolin Wang, Binliang Zhang, Zunhua Yang, Wei Li, Wufu Zhu, Shan Xu

**Affiliations:** 1 School of Pharmacy, Jiangxi Science & Technology Normal University, Nanchang 330013, Jiangxi, China; zhaobb_0628@163.com (B.Z.); m13767032532@163.com (F.L.); wangcllw@163.com (C.W.); zbl1045762244@163.com (B.Z.); 2 School of Pharmacy, Jiangxi University of Traditional Chinese Medicine, Nanchang 330004, Jiangxi, China; mtdzcool@163.com; 3 School of Chemistry and Bioengineering, Yichun University, Yichun 336000, Jiangxi, China; nobel2025@163.com

**Keywords:** 4-phenylaminoquinoline, phenylsulfonylurea, PI3K/mTOR, inhibitor

## Abstract

Five series of novel phenylsulfonylurea derivatives, **19a**–**d**, **20a**–**d**, **21a**–**d**, **22a**–**d** and **23a**–**d**, bearing 4-phenylaminoquinoline scaffold were designed, synthesized and their IC_50_ values against four cancer cell lines (HepG-2, A549, PC-3 and MCF-7) were evaluated. Most compounds showed moderate cytotoxicity activity against the cancer cell lines. Structure–activity relationships (SARs) and pharmacological results indicated that introduction of 4-aminoquinoline scaffold and phenylsulfonylurea scaffold were beneficial for anti-tumor activity. Moreover, para-methoxyl substitution of 4-anilino moiety and para-halogen substitution of phenylsulfonylurea have different impacts on different series of compounds. Furthermore, the micromolecule group substitution in the 6-position of the quinoline ring have a slight impact on the cellular activity of the target compounds.

## 1. Introduction

**Figure 1:**
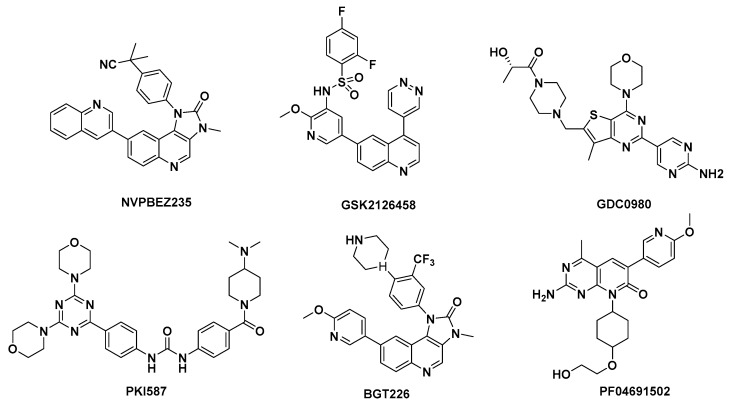
Structures of representative clinical PI3K/mTOR dual inhibitors.

PI3K (phosphatidylinositol-3-kinase) is a kind of cellular signaling molecule, which is closely associated with human cancers. According to the primary structures, regulatory mechanisms and distribution positions, the PI3K families [1,2,3] are divided into four different types: Type I, Type II, Type III and Type IV. The type I PI3K enzymes are further divided into PI3Kα, PI3Kβ, PI3Kδ and PI3Kγ [4,5]. PI3K/mTOR signaling pathway plays a crucial role in regulating various cellular physiological activities such as proliferation, growth, proliferation and apoptosis [6]. With the development of cytobiology, accumulating studies have shown that the PI3K/mTOR signaling pathway is frequently disregarded in most tumor cells, leading to malignant proliferation of normal organism cells [7,8,9]. In addition, research has also shown that PI3K/mTOR signaling pathway can promote the occurrence of cancer by promoting angiogenesis, accelerating cell growth cycle and promoting tumor cell metastasis [10]. Therefore, the development of double inhibitors targeting PI3K/mTOR has become a hotspot in this field. Thus far, numerous PI3K/mTOR double target inhibitors have been developed into clinical trial, such as NVPBEZ235 [11], GSK2126458 [12], GDC0980 [13], PKI587 [14], BGT226 [15] and PF06491502 [16] (Figure 1). Furthermore, most of these compounds shared quinoline core and exhibited remarkable potency in both enzymatic and cellular inhibitory activity [17]. Research data further demonstrated that PI3K/mTOR double target inhibitors have a great potential in the process of anti-tumor therapy [18,19].

4-Amino quinazoline derivatives have been widely used in small molecule antitumor drugs for their multiple biological activities, especially EGFR inhibitors [17]. Compounds containing 4-amino quinazoline core account for a substantial proportion of therapeutic drugs [20], such as Gefitinib, Erlotinib, Afatinib and Vandetanib. The potency of these drugs has been widely recognized in clinical treatment. Accordingly, we introduced 4-amino quinazoline structure into PI3K/mTOR inhibitor to investigate antiproliferative activity of 4-amino quinazoline structure on tumor cells.

**Figure 2:**
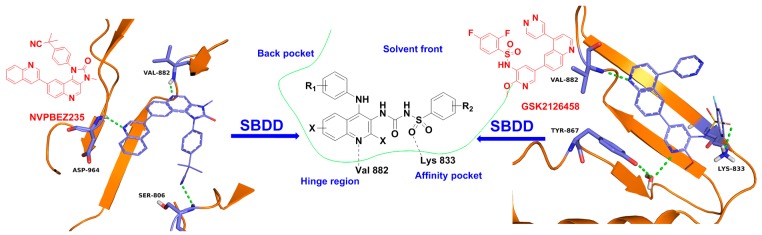
Structure based drug design strategy based on the molecular simulation.

We firstly introduced 4-aminoquinoline structure by ring-opening the imidazolone structure of NVPBEZ235. To guide our design, we carried out the molecular simulation to find the differences between the binding mode of GSK2126458 and NVPBEZ235 with PI3Kγ protein. From the docking results in Figure 2, we can see that both drugs can form the hydrogen bond between the N-atom in quinoline rings and valine. The -CN of substituted aniline side chain in the NVPBEZ235 points to the hydrophobic cavity and forms a hydrogen bond with SER806. This suggests that the para position of 4-aniline side chain is a key active site. Therefore, we introduced electron-withdrawing group –Br, electron-donating group -OCH_3_, 3-*Cl*-4-*F*-aniline active group, 4-*Br*-2-*F*-aniline active group and retained -CN to change benzene ring electron cloud density. Through this design strategy, we expect to obtain desirable inhibitors with better PI3K/mTOR inhibition activity. In addition, benzene sulfonamide structure of GSK2124658 buried into hydrophobic pockets, and the O-atom of sulfonyl group formed a hydrogen bond with residue LYS833. Therefore, we secondly introduced benzene sulfonamide group into NVPBEZ235 to explore potential interactions between the PI3Kγ protein and target compounds, and also improve the water solubility. Based on the above, we used the same design strategy to introduce different substituent groups into the para-position of phenylsulfonylurea. On the one hand, we changed the electron cloud density of benzene ring. On the other hand, we appropriately increased the space structure of benzene ring to enhance the affinity of small molecule ligand with receptor protein. Finally, series of 4-aniline quinoline derivatives containing phenylsulfonylurea structure were designed, synthesized and all compounds were evaluated for their anti-proliferative effects.

## 2. Results and Discussion

### 2.1. Chemistry

**Scheme 1:**
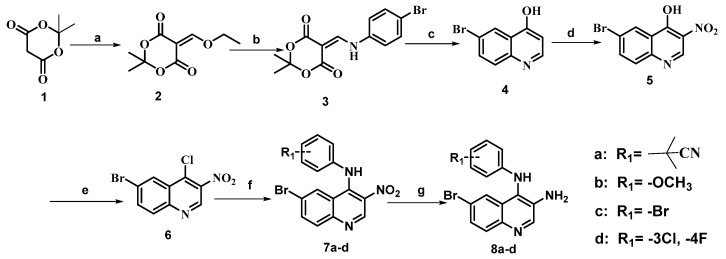
The synthetic route for target compounds **8a**–**d**. Reagents and conditions: (a) Triethoxy methane; (b) 4-Bromoanilines, C_2_H_5_OH, reflux, 105 °C. 6 h; (c) Ph_2_O, 200 °C, Microwave, 15 min; (d) HNO_3_, Propionic acid, 125 °C, 2 h; (e) POCl_3_, DMF, 100 °C, 3 h; (f) Substituted aniline, Acetic acid, 100 °C, 5 h; and (g) FeCl_3_, N_2_H_4_·H_2_O, C_2_H_5_OH, 80 °C, 3 h.

**Scheme 2:**
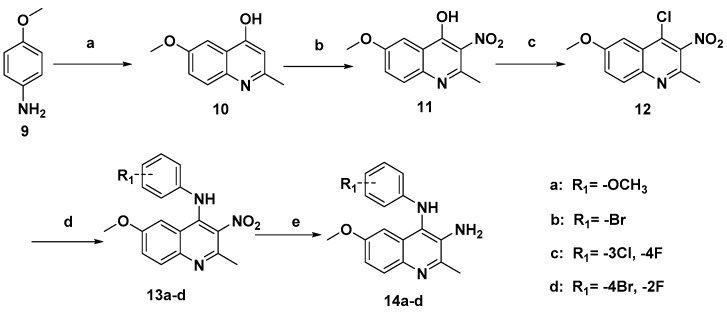
The synthetic route for target compounds **13a**–**d**. Reagents and conditions: (a) PPA, 190 °C, 6 h; (b) HNO_3_, Propionic acid, 125 °C, 2 h; (c) POCl_3_, DMF, 100 °C, 3 h; (d) Substituted aniline, Acetic acid, 100 °C, 5 h; and (e) FeCl_3_, N_2_H_4_·H_2_O, C_2_H_5_OH, 80 °C, 3 h.

**Scheme 3:**
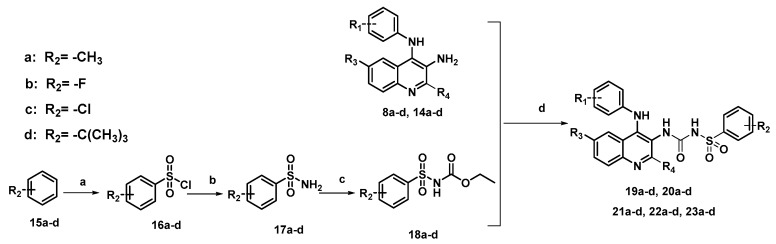
The synthetic route for target compounds **19a**–**d**, **20a**–**d**, **21a**–**d**, **22a**–**d** and **23a**–**d**. Reagents and conditions: (a) HSO_3_Cl, reflux, 6 h; (b) C_2_H_5_OH, 0 °C, 1 h; (c) DIPEA, CH_2_Cl_2_, r.t., 0.5 h; and (d) Toluene, 120 °C, 6 h.

Based on the structure based drug design (SBDD) strategy, taking NVPBEZ235 as reference compound, five series of PI3K/mTOR inhibitors were designed and synthesized. The synthetic routes of target compounds **19a**–**d**, **20a**–**d**, **21a**–**d**, **22a**–**d** and **23a**–**d** are outlined in Scheme 1, Scheme 2 and Scheme 3.

As shown in Scheme 1, Scheme 2 and Scheme 3, all target compounds were synthesized by resolution synthetic method. Target compounds were separately split into 4-anilinoquinoline fragment in the left half and phenylsulfonylurea fragment in the right half, and finally the two fragments were connected to obtain target compounds. As shown in Scheme 1, we used commercially available isopropyl malonate (**1**) and triethyl orthoformate as starting materials to obtain segments **8a**–**d** by seven steps of condensation, cyclization, substitution, nitration, chlorination, amination and reduction. Then, we used **9** as the starting material to get fragments **14a**–**d** by a method similar to that of synthesizing **8a**–**d**. Detailed synthetic route of **14a**–**d** is shown in Scheme 2. Next, as show in Scheme 3, we used different substituted aromatic rings as starting materials to obtain fragments **18a**–**d** by chlorosulfonation, amminolysis and substitution reaction. Finally, target compounds **19a**–**d**, **20a**–**d**, **21a**–**d**, **22a**–**d** and **23a**–**d** were obtained by linking fragments **18a**–**d** with fragments **8a**–**d** and **14a**–**d**, respectively. The structural information of all target compounds was confirmed by ^1^H-NMR, ^13^C-NMR and TOF MS (ES+), which were in agreement with the structures depicted.

### 2.2. Biological Discussion

Four cells (HepG-2, A549, PC-3, MCF-7) were selected to evaluate the in vitro antiproliferative activity of all target compounds. As shown in Table 1, phenylsulfonylurea derivatives (**19a**–**d**, **20a**–**d**, **21a**–**d** and **22a**–**d**) with bromine substitution at 6-position of quinoline ring showed moderate antiproliferative activity against HepG-2 cell, A549 cell and PC-3 cell, and some compounds (**19a** and **19c**) showed strong sensitivity to MCF-7 cell. In addition, the introduction of different substituents into 4-position of aniline in the quinoline ring can significantly improve anti-proliferative activity and cell selectivity of target compounds, and when aniline was substituted with -CN or -OCH_3_, target compounds (**19a**–**d** and **20a**–**d**) showed stronger anti-proliferative activity to MCF-7 cell. Compounds with -CN or -OCH_3_ substituted at the 4-position of aniline structure in the quinoline ring showed better anti-proliferative activity against MCF-7 cell than that of the other three cells. When -CH_3_ or Cl-atom were introduced into the phenylsulfonylurea structure, target compounds (**20c** and **22b**) showed good anti-proliferative activity on MCF-7 cell, but when aniline was substituted with F-atom or Cl-atom, the anti-proliferative activity of target compound (**21a**–**d**) on MCF-7 cell was weaker than that of other three cells. Overall, 4-anilino side chain para-methoxy substituted compounds **20a**–**d** showed better MCF-7 cell anti-proliferation activity than other compounds (**19a**–**d**, **21a**–**d** and **22a**–**d**). We speculate that electron-donating group -OCH_3_ increased the electron cloud density of aniline side chain. Thus, the force between aniline side chain and hydrophobic cavity of protein was enhanced.

**Figure 3:**
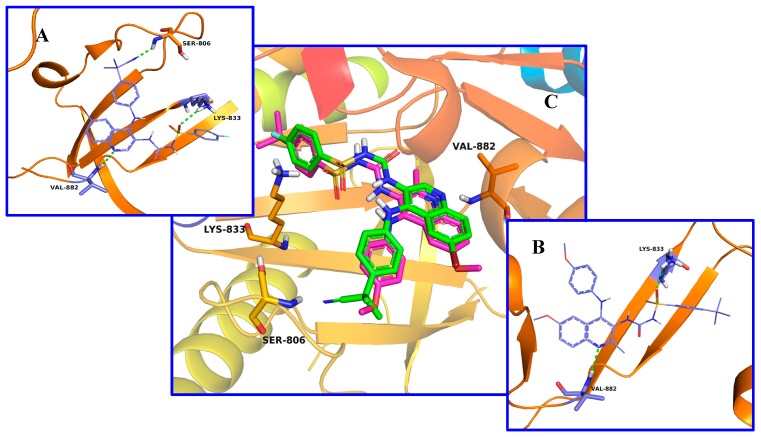
(**A**) The binding mode of compound **19a** with PI3Kγ (3L08) kinase; (**B**) the binding mode of compound **23b** with PI3Kγ kinase; and (**C**) the contrast of binding mode between compound **19a** (green) and **23b** (red).

Phenylsulfonylurea derivatives (**23a**–**d**) with methoxyl substitution at 6-position of the quinoline ring showed moderate antitumor activity against HepG-2 cell, A549 cell and PC-3 cell. Similarly, 4-anilino side chain para-methoxy substituted compounds **23a** and **23b** showed better MCF-7 cell anti-proliferation activity than that of other compounds (**23c** and **23d**). It is noteworthy that the activity of compounds **23a**–**d** to HepG-2 cell was generally superior to that of the other three cells, especially compounds **23b** and **23d** (with an IC_50_ value of 2.71 µM and 6.08 µM, respectively). We supposed that the quinoline ring and the 4-phenylamino side chain of compound **23b** are not only both located in the hydrophobic cavity of the protein but also all substituted by electron-donating group -OCH_3_ group, which greatly increased the electron cloud density of the structure in the left half of compound **23b.** Thus, the affinity between compound **23b** and the target protein was enhanced. In addition, from the docking results (Figure 3), we can see that the -OCH_3_ group on the quinoline ring of compound **23b** points to inside of the protein cavity and fully occupies the space of this part, thus resulting in better antiproliferative activity of compounds **23a**–**d** against HepG-2 cell.

Finally, the inhibitory rate against PI3Kα kinase and mTOR kinase of selected compounds **19a** and **23b** at 10 µM was further examined. As shown in Table 2, enzymatic activity results of compounds **19a** and **23b** exhibited a moderate inhibitory rate against PI3Kα kinase and mTOR kinase.

### 2.3. Molecular Docking Study

To explore the binding modes of target compounds (**19a** and **23b**) with the active site of PI3Kγ, molecular docking simulation studies were carried out by the AutoDock 4.2 software. The docking tutorial we used and the detailed AutoDock basic operational methods can be found at: http://autodock.scripps.edu/faqs-help/tutorial. Based on in vitro inhibition results, we selected compounds **19a** and **23b** as ligand examples, and the structures of PI3Kγ (PDB code: 3L08) were selected as the docking models. Only the best-scoring ligand–protein complex (Figure 3) was used for the binding site analysis.

The binding mode of compounds **19a** and **23b** with the active site of PI3Kγ molecular is shown in Figure 3, which depicts that the oxygen atom on benzene sulfonamide group of compounds **19a** and **23b** both formed a hydrogen bond with PI3Kγ residue LYS833 and the nitrogen atom in quinoline ring formed a hydrogen bond with hinge residue VAL882. In the docking model of compound **19a** with PI3Kγ, we can see the cyano group on the aniline group in the 4-position of quinoline ring formed a hydrogen bond with the residue SER806. All these factors above contribute to the excellent antiproliferative activity and selectivity of the compound **19a.** The whole 4-aminoquinoline ring structure of the selected compounds extended into ATP hydrophobic pocket, which is basically the same as our previous prediction binding mode (show in Figure 2). Furthermore, binding modes of compounds **19a** and **23b** were almost completely overlapped. The abovementioned SAR (Structure–Activity Relationship) analysis and molecular docking study results may allow the rational design of potential PI3K and mTOR inhibitors.

## 3. Experimental Section

### 3.1. General Information

Unless otherwise required, all reagents used in the experiment were purchased as commercial analytical grade and used without further purification. Frequently used solvents (Ethanol, petroleum ether, ethyl acetate, dichloromethane, etc.) were absolutely anhydrous. All actions were monitored through GF_254_ thin-layer chromatography plate and spots were visualized with iodine or light (in 254 nm or 365 nm). The structure of the target compound was confirmed by ^1^H-NMR and ^13^C-NMR spectra at room temperature on Bruker 400 MHz spectrometer (Bruker Bioscience, Billerica, MA, USA) with tetramethylsilane (TMS) as an internal standard. Mass spectrometry (MS) was performed on Waters High Resolution Quadrupole Time of Flight Tandem Mass Spectrometry (QTOF). The purity of the compound was determined by Agilent 1260 liquid chromatograph fitted with an Inertex-C18 column. All target compounds had the purity of ≥95%.

### 3.2. Chemistry

#### 3.2.1. General Procedure for the Preparation of Compounds **8a**–**d**, **14a**–**d** and **18a**–**d**

We using isopropyl malonate (**1**) and triethyl orthoformate as the starting material to get intermediate **2**. Next, intermediate **2** was condensed and cyclized with p-bromoaniline to obtain 4-hydroxy-6-bromoquinoline (**5**), and then 3-nitro-4-chloro-6-bromoquinoline (**6**) was generated by nitration and chlorination. Intermediate **6** reacted with para-substituted anilines (**a**–**d**) to obtain intermediates **7a**–**d**, and then underwent a reduction reaction to obtain intermediates **8a**–**d**. Similarly, we used 4-methoxyaniline (**9**) as the starting material to obtain intermediates **14a**–**d** through a similar reaction. Chlorosulfonic acid was reacted with different substituted anilines (**15a**–**d**) to obtain (**16a**–**d**), and then 1**8a**–**d** were obtained by amination reaction and acylation reaction.

#### 3.2.2. General Procedure for Preparation of Target Compounds **19a**–**d**, **20a**–**d**, **21a**–**d**, **22a**–**d** and **23a**–**d**

A solution of ethyl (phenylsulfonyl) carbamate (1.2 mmol, **18a**–**d**) in toluene (5 mL) was added dropwise to a solution of **8a**–**d** or **14a**–**d** (1 mmol) in toluene (15 mL). Upon completion of the addition, the reaction mixture was stirred at 120 °C for 6 h and monitored by thin-layer chromatography (TLC). The reaction mixture was cooled to room temperature and concentrated under a reduced pressure. Then, ethyl acetate was added and the insoluble materials were collected by filtration and dried to yield the target compounds **19a**–**d**, **20a**–**d**, **21a**–**d**, **22a**–**d** and **23a**–**d**, which were recrystallized from isopropanol.

*N-((6-Bromo-4-((4-(2-cyanopropan-2-yl)phenyl)amino)quinolin-3-yl)carbamoyl)-4-fluorobenzenesulfonamide carboxamide* (**19a**) A pale white solid; Yield: 52.6%; m.p.: 315.2–316.4 °C; ^1^H-NMR (400 MHz, DMSO-*d*_6_) δ (ppm): 12.01 (s, 1H), 8.95 (s, 1H), 8.08 (d, *J* = 9.0 Hz, 1H), 8.05–7.94 (m, 4H), 7.82 (dd, *J* = 16.8, 8.6 Hz, 3H), 7.59–7.50 (m, 3H), 7.11 (s, 1H), 1.95 (s, 6H). ^13^C NMR (400 MHz, DMSO-*d*_6_) δ 165.41, 153.89, 143.83, 142.85, 134.90, 132.57, 130.12, 129.89 × 2, 129.42, 129.06, 128.97, 127.26 × 2, 124.80, 122.84, 122.66, 119.37, 116.55 × 2, 116.40, 116.33 × 2, 37.32, 28.86 × 2. TOF MS ES^+^ (*m*/*z*): [M + H]^+^, calcd for C_26_H_21_BrFN_5_O_3_S: 581.0533, found: 582.0611.

*N-((6-Bromo-4-((4-(2-cyanopropan-2-yl)phenyl)amino)quinolin-3-yl)carbamoyl)-4-chlorobenzenesulfonamide* (**19b**). A pale white solid; Yield: 50.1%; m.p.: 279.2–280.1 °C; ^1^H-NMR (400 MHz, DMSO-*d*_6_) δ (ppm): 11.87 (s, 1H), 8.81 (s, 1H), 8.23(d, *J* = 9.8Hz, 2H), 7.94 (d, *J* = 9.0 Hz, 1H), 7.89 (s, 1H), 7.88 (d, *J* = 3.1 Hz, 1H), 7.85 (s, 1H), 7.83 (s, 1H), 7.70 (d, *J* = 8.2 Hz, 2H), 7.66 (d, *J* = 9.1 Hz, 1H), 7.45–7.38 (m, 3H), 6.97 (s, 1H), 1.81 (s, 6H), ^13^C-NMR (400 MHz, DMSO-*d*_6_) δ 153.88, 143.79, 143.12, 135.17 × 2, 134.93, 132.86 × 2, 129.95, 129.90 × 2, 129.15, 127.24 × 2, 126.09, 124.80, 122.83, 122.62 × 2, 122.62, 119.30, 116.45, 102.00, 37.32, 28.86 × 2, 21.37. TOF MS ES^+^ (*m*/*z*): [M + H]^+^, calcd for C_26_H_21_BrClN_5_O_3_S: 597.0237, found: 598.0315.

*N-((*6*-Bromo-4-((4-(2-cyanopropan-2-yl)phenyl)amino)quinolin-3-yl)carbamoyl)-4-methylbenzenesulfonamide* (**19c**). A pale white solid; Yield: 51.4%; m.p.: 289.2–290.4 °C; ^1^H-NMR (400 MHz, DMSO-*d*_6_) δ (ppm): 12.01 (s, 1H), 8.95 (s, 1H), 8.25(d, *J* = 9.5 Hz), 8.08 (d, *J* = 9.0 Hz, 1H), 7.98 (d, *J* = 8.4 Hz, 3H), 7.84 (d, *J* = 8.2 Hz, 2H), 7.80 (d, *J* = 9.1 Hz, 1H), 7.59–7.51 (m, 3H), 7.11 (s, 1H), 6.23(s, 1H), 2.30(s, 3H) 1.95 (s, 6H). ^13^C-NMR (400 MHz, DMSO-*d*_6_) δ 153.88, 143.79, 143.12, 135.17 × 2, 134.93, 132.86 × 2, 129.95, 129.90 × 2, 129.15, 127.24 × 2, 126.09, 124.80, 122.83, 122.62 × 2, 122.62, 119.30, 116.45, 102.00, 37.32, 28.86 × 2, 21.37. TOF MS ES^+^ (*m*/*z*): [M + H]^+^, calcd for C_27_H_24_BrN_5_O_3_S: 577.0783, found: 578.0861.

*N-((6-Bromo-4-((4-(2-cyanopropan-2-yl)phenyl)amino)quinolin-3-yl)carbamoyl)-4-(tert-butyl)benzenesulfonamide* (**19d**). A pale white solid; Yield: 45.6%; m.p.: 287.2–288.4 °C; ^1^H-NMR (400 MHz, DMSO-*d*_6_) δ (ppm): 12.06 (s, 1H), 9.00 (s, 1H), 8.23(s, 1H), 8.13 (d, *J* = 9.0 Hz, 1H), 8.03 (d, *J* = 8.4 Hz, 4H), 7.89 (d, *J* = 8.2 Hz, 2H), 7.85 (d, *J* = 9.1 Hz, 1H), 7.65–7.57 (m, 3H), 7.16 (s, 1H), 2.00 (s, 1H), 1.63(s, 9H), ^13^C-NMR (400 MHz, DMSO-*d*_6_) δ 156.15, 153.88, 143.77, 143.09, 135.17, 134.91, 132.87, 129.96, 129.92, 128.97 × 3, 127.25 × 2, 125.32 × 2, 121.18, 121.09, 120.15 × 2, 119.72, 118.38, 116.44, 37.32, 29.72, 28.84 × 3, 25.62 × 2. TOF MS ES^+^ (*m*/*z*): [M + H]^+^, calcd for C_30_H_30_BrN_5_O_3_S: 619.1253, found: 620.1331.

*N-((6-Bromo-4-((4-methoxyphenyl)amino)quinolin-3-yl)carbamoyl)-4-fluorobenzenesulfonamide* (**20a**). A pale white solid; Yield: 47.9%; m.p.: 289.1–290.2 °C; ^1^H-NMR (400 MHz, DMSO-*d*_6_) δ (ppm): 11.93 (s, 1H), 8.93 (s, 1H), 8.08 (d, *J* = 9.0 Hz, 1H), 7.86 (d, *J* = 8.0 Hz, 2H), 7.82–7.77 (d, *J* = 8.6 Hz, 1H), 7.68 (d, *J* = 8.7 Hz, 2H), 7.52 (d, *J* = 8.0 Hz, 2H), 7.43 (s, 2H), 7.37 (d, *J* = 8.7 Hz, 2H), 7.28 (s, 1H), 4.05 (s, 3H), ^13^C-NMR (400 MHz, DMSO-*d*_6_) δ 165.72, 153.88, 152.52, 143.78, 138.17, 136.91, 132.86, 130.98 × 2, 129.92, 128.81, 128.26, 121.98, 121.61 × 2, 119.57, 118.32, 117.44, 116.02 × 2, 115.18 × 2, 59.84. TOF MS ES^+^ (*m*/*z*): [M + H]^+^, calcd for C_23_H_18_BrFN_4_O_4_S: 544.0216, found: 545.0294.

*N-((6-Bromo-4-((4-methoxyphenyl)amino)quinolin-3-yl)carbamoyl)-4-chlorobenzenesulfonamide* (**20b**). A pale white solid; Yield:48.2%; m.p.: 282.2–283.1 °C; ^1^H-NMR (400 MHz, DMSO-*d*_6_) δ (ppm): 10.83 (s, 1H), 8.87 (s, 1H), 8.68 (s, 1H), 8.48 (s, 1H), 8.08-7.89 (d, *J* = 8.0 Hz, 2H), 7.84 (d, *J* = 8.0 Hz, 2H), 7.58 (d, *J* = 8.7 Hz, 2H), 7.51 (d, *J* = 8.0 Hz, 2H), 7.43 (s, 2H), 7.37 (d, *J* = 8.7 Hz, 2H), 7.07 (s, 1H), 3.97 (s, 3H), ^13^C-NMR (400 MHz, DMSO-*d*_6_) δ 153.72, 153.03, 144.05, 138.95, 138.47, 137.41, 132.86, 130.03 × 3, 128.92 × 2, 128.81, 128.02, 121.88, 121.76 × 2, 119.86, 118.91, 117.84, 115.11 × 2, 59.64. TOF MS ES^+^ (*m*/*z*): [M + H]^+^, calcd for C_23_H_18_BrClN_4_O_4_S: 559.9921, found: 560.9999.

*N-((6-Bromo-4-((4-methoxyphenyl)amino)quinolin-3-yl)carbamoyl)-4-methylbenzenesulfonamide* (**20c**). A pale white solid; Yield: 46.6%; m.p.: 279.2–280.6 °C; ^1^H-NMR (400 MHz, DMSO-*d*_6_) δ (ppm): 11.77 (s, 1H), 8.77 (s, 1H), 7.93 (d, *J* = 9.0 Hz, 1H), 7.70 (d, *J* = 8.0 Hz, 2H), 7.67–7.60 (m, 1H), 7.53 (d, *J* = 8.7 Hz, 2H), 7.36 (d, *J* = 8.0 Hz, 2H), 7.28 (s, 2H), 7.21 (d, *J* = 8.7 Hz, 2H), 7.13 (s, 1H), 3.89 (s, 3H), 2.37 (s, 3H). ^13^C-NMR (400 MHz, DMSO-*d*_6_) δ 160.46, 154.20, 143.08, 142.33, 141.89, 135.04, 132.80, 130.57 × 2, 129.91, 129.77, 129.49 × 2, 127.81, 126.08 × 2, 122.63, 122.59, 119.24, 116.57, 115.52 × 2, 56.13, 21.38. TOF MS ES^+^ (*m*/*z*): [M + H]^+^, calcd for C_24_H_21_BrN_4_O_4_S: 540.0467, found: 541.0545.

*N-((6-Bromo-4-((4-methoxyphenyl)amino)quinolin-3-yl)carbamoyl)-4-(tert-butyl)benzenesulfonamide* (**20d**). A pale white solid; Yield: 40.6%; m.p.: 263.3–264.2 °C; ^1^H-NMR (400 MHz, DMSO-*d*_6_) δ (ppm): 11.93 (s, 1H), 8.93 (s, 1H), 8.08 (d, *J* = 9.0 Hz, 1H), 7.86 (d, *J* = 8.0 Hz, 2H), 7.82–7.77 (d, *J* = 8.6 Hz, 1H), 7.68 (d, *J* = 8.7 Hz, 2H), 7.52 (d, *J* = 8.0 Hz, 2H), 7.43 (s, 2H), 7.37 (d, *J* = 8.7 Hz, 2H), 7.28 (s, 1H), 4.05 (s, 3H),1.72(s, 9H), ^13^C-NMR (400 MHz, DMSO-*d*_6_) δ 154.87, 153.23, 152.12, 143.27, 138.54, 137.24, 132.48, 129.45, 128.36, 128.97 × 2, 129.87, 125.32 × 2, 121.89, 121.43 × 2, 119.58, 118.65, 118.47, 115.52 × 2, 55.98, 35.12, 31.25 × 3. TOF MS ES^+^ (*m*/*z*): [M + H]^+^, calcd for C_27_H_27_BrN_4_O_4_S: 582.0936, found: 583.1015.

*N-((6-Bromo-4-((4-bromophenyl)amino)quinolin-3-yl)carbamoyl)-4-fluorobenzenesulfonamide* (**21a**). A pale white solid; Yield: 45.8%; m.p.: 278.7–279.5 °C; ^1^H-NMR (400 MHz, DMSO-*d*_6_) δ (ppm): 12.04 (s, 1H), 8.95 (s, 1H), 8.84 (s, 2H), 8.76 (s, 1H), 8.34 (d, *J* = 12.0 Hz, 4H), 8.19 (s, 1H), 7.76 (d, *J* = 8.5 Hz, 2H), 7.42 (d, *J* = 8.5 Hz, 2H), 6.60 (s, 1H). ^13^C-NMR (400 MHz, DMSO-*d*_6_) δ 144.98, 138.52, 135.18, 133.35, 131.99, 131.92, 131.73, 131.54, 131.05, 130.95, 129.06, 128.97, 128.45, 125.57, 123.84, 120.28, 118.54 × 2, 117.76, 116.51, 116.36 × 2. TOF MS ES^+^ (*m*/*z*): [M + H]^+^, calcd for C_22_H_15_Br_2_FN_4_O_3_S: 591.9216, found: 592.9294.

*N-((6-Bromo-4-((4-bromophenyl)amino)quinolin-3-yl)carbamoyl)-4-chlorobenzenesulfonamide* (**21b**). A pale white solid; Yield: 42.6%; m.p.: 284.1–285.4 °C; ^1^H-NMR (400 MHz, DMSO-*d*_6_) δ (ppm): 11.88 (s, 1H), 8.79 (s, 1H), 8.60 (s, 1H), 8.18 (d, *J* = 12.0 Hz, 4H), 8.03 (s, 1H), 7.94 (d, *J* = 8.2 Hz, 1H), 7.93–7.90 (m, 2H), 7.45 (s, 1H), 7.26 (d, *J* = 8.5 Hz, 2H), 6.44 (s, 1H). ^13^C-NMR (400 MHz, DMSO-*d*_6_) δ 144.63, 138.07, 134.75, 132.89, 132.46, 131.54 × 2, 131.34, 131.05, 129.56, 129.07 × 2, 128.04, 127.62 × 2, 127.02, 123.39, 122.07, 119.82, 118.96, 116.03 × 2. TOF MS ES^+^ (*m*/*z*): [M + H]^+^, calcd for C_22_H_15_Br_2_ClN_4_O_3_S: 607.8920, found: 608.8998.

*N-((6-Bromo-4-((4-bromophenyl)amino)quinolin-3-yl)carbamoyl)-4-methylbenzenesulfonamide* (**21c**). A pale white solid; Yield: 41.9%; m.p.: 279.6–280.2 °C; ^1^H-NMR (400 MHz, DMSO-*d*_6_) δ (ppm): 12.01 (s, 1H), 8.92 (s, 1H), 8.76 (d, *J* = 11.1 Hz, 4H), 8.13 (s, 1H), 7.94 (s, 2H), 7.74 (d, *J* = 8.3 Hz, 1H), 7.49 (d, *J* = 7.8 Hz, 3H), 7.25 (s, 1H), 6.58 (s, 1H), 2.49 (s, 3H). ^13^C-NMR (400 MHz, DMSO-*d*_6_) δ 144.71, 144.21, 141.87, 141.46, 138.11, 132.89, 131.55 × 2, 131.41, 131.07, 129.30 × 2, 128.08, 126.99, 125.64 × 2, 123.37, 120.88, 119.82, 116.00 × 2, 108.86, 20.91. TOF MS ES^+^ (*m*/*z*): [M + H]^+^, calcd for C_23_H_18_Br_2_N_4_O_3_S: 587.9466, found: 588.9545.

*N-((6-Bromo-4-((4-bromophenyl)amino)quinolin-3-yl)carbamoyl)-4-(tert-butyl)benzenesulfonamide* (**21d**). A pale white solid; Yield: 39.3%; m.p.: 259.4–260.3 °C; ^1^H-NMR (400 MHz, DMSO-*d*_6_) δ (ppm): δ 11.88 (s, 1H), 8.68 (s, 2H), 8.60 (s, 1H), 8.18 (d, *J* = 12.0 Hz, 3H), 8.03 (s, 1H), 7.26 (d, *J* = 8.5 Hz, 2H), 7.18-7.09 (m, 4H), 6.44 (s, 1H), 1.63(s, 9H). ^13^C-NMR (400 MHz, DMSO-*d*_6_) δ 155.20, 145.17, 144.65, 141.80, 141.03, 138.58, 133.35, 132.00 × 2, 131.88, 131.54, 128.53, 127.44, 126.18 × 2, 125.98 × 2, 123.80, 121.28, 120.28, 116.43 × 2, 109.28, 35.23, 31.32 × 2. TOF MS ES^+^ (*m*/*z*): [M + H]^+^, calcd for C_26_H_24_Br_2_N_4_O_3_S: 629.9936, found: 631.0014.

*N-((6-Bromo-4-((3-chloro-4-fluorophenyl)amino)quinolin-3-yl)carbamoyl)-4-fluorobenzenesulfonamide* (**22a**). A pale white solid; Yield: 46.5%; m.p.: 295.1–296.3 °C; ^1^H-NMR (400 MHz, DMSO-*d*_6_) δ (ppm): 11.87 (s, 1H), 8.34 (s, 1H), 7.81 (d, *J* = 5.8 Hz, 1H), 7.77–7.59 (m,2H), 7.50 (d, *J* = 8.5 Hz, 2H), 7.18 (q, *J* = 9.1 Hz, 2H), 7.01 (dd, *J* = 18.2, 8.8 Hz, 2H), 6.95–6.90 (m, 1H), 6.04 (d, *J* = 2.2 Hz, 1H). ^13^C-NMR (400 MHz, DMSO-*d*_6_) δ 156.47, 153.39, 142.65, 134.77 × 2, 132.51 × 2, 132.10, 131.50 × 2, 130.15, 129.56 × 2, 128.44, 122.37, 122.01 × 2, 120.65, 119.02, 118.15, 117.92, 115.98. TOF MS ES^+^ (*m*/*z*): [M + H]^+^, calcd for C_22_H_14_BrClF_2_N_4_O_3_S: 565.9627, found: 566.9705.

*N-((6-Bromo-4-((3-chloro-4-fluorophenyl)amino)quinolin-3-yl)carbamoyl)-4-chlorobenzenesulfonamide* (**22b**). A pale white solid; Yield: 42.6%; m.p.: 282.3–283.7 °C; ^1^H-NMR (400 MHz, DMSO-*d*_6_) δ (ppm): 11.91 (s, 1H), 10.48 (s, 1H), 7.84 (d, *J* = 8.0 Hz, 3H), 7.76 (d, *J* = 7.5 Hz, 2H), 7.68 (d, *J* = 8.3 Hz, 1H), 7.60 (dd, *J* = 18.5, 10.8 Hz, 2H), 7.54 (d, *J* = 7.9 Hz, 2H), 7.28 (d, *J* = 9.1 Hz, 1H), 6.29 (s, 1H). ^13^C-NMR (400 MHz, DMSO-*d*_6_) δ 153.83, 143.10, 135.27, 132.96, 132.55, 131.93 × 2, 130.58, 130.50, 130.01, 129.51, 128.89, 128.13 × 2, 128.08, 127.96, 122.86, 122.46, 119.46, 118.59, 118.37, 116.45. TOF MS ES^+^ (*m*/*z*): [M + H]^+^, calcd for C_22_H_14_BrCl_2_FN_4_O_3_S: 581.9331, found: 582.9409.

*N-((6-Bromo-4-((3-chloro-4-fluorophenyl)amino)quinolin-3-yl)carbamoyl)-4-methylbenzenesulfonamide* (**22c**). A pale white solid; Yield: 43.2%; m.p.: 278.7–279.9 °C; ^1^H-NMR (400 MHz, DMSO-*d*_6_) δ (ppm): 11.77 (s, 1H), 10.34 (s, 1H), 7.69 (d, *J* = 8.0 Hz, 3H), 7.61 (d, *J* = 7.5 Hz, 2H), 7.54 (d, *J* = 8.3 Hz, 1H), 7.46 (d, *J* = 7.7 Hz, 2H), 7.39 (d, *J* = 7.9 Hz, 2H), 7.13 (d, *J* = 9.1 Hz, 1H), 6.14 (s, 1H), 2.68 (s, 3H). ^13^C-NMR (400 MHz, DMSO-*d*_6_) δ 153.84, 143.08 × 2, 135.23 × 2, 132.96 × 2, 131.95 × 2, 130.62, 130.54, 130.04 × 2, 128.90, 122.81, 122.45, 121.10, 120.91, 119.48, 118.61, 118.39, 116.43, 21.4. TOF MS ES^+^ (*m*/*z*): [M + H]^+^, calcd for C_23_H_17_BrClFN_4_O_3_S: 561.9877, found: 562.9956.

*N-((6-Bromo-4-((3-chloro-4-fluorophenyl)amino)quinolin-3-yl)carbamoyl)-4-(tert-butyl)benzenesulfonamide* (**22d**). A pale white solid; Yield: 38.7%; m.p.: 290.1–291.6 °C; ^1^H-NMR (400 MHz, DMSO-*d*_6_) δ (ppm): 11.83 (s, 1H), 10.40 (s, 1H), 7.90 (d, *J* = 7.3 Hz, 3H), 7.75 (d, *J* = 8.0 Hz, 3H), 7.67 (d, *J* = 7.5 Hz, 2H), 7.52 (d, *J* = 7.7 Hz, 1H), 7.45 (d, *J* = 7.9 Hz, 1H), 6.66 (s, 1H), 2.65 (s, 3H), 1.86 (s, 9H). ^13^C-NMR (400 MHz, DMSO-*d*_6_) δ 153.85, 143.08, 135.23, 132.96, 132.51, 131.95, 130.62, 128.41, 128.01 × 3, 122.82, 122.46, 121.5 × 2, 119.49, 118.61 × 2, 118.39 × 2, 116.43, 34.3, 31.4 × 3. TOF MS ES^+^ (*m*/*z*): [M + H]^+^, calcd for C_26_H_23_BrClFN_4_O_3_S: 604.0347, found: 605.0425.

*4-Chloro-N-((6-methoxy-4-((4-methoxyphenyl)amino)-2-methylquinolin-3-yl)carbamoyl)benzenesulfonamide* (**23a**). A pale white solid; Yield: 42.6%; m.p.: 290.8–291.2 °C; ^1^H-NMR (400 MHz, DMSO-*d*_6_) δ (ppm): 11.86 (s, 1H), 8.31(d, *J* = 10.8 Hz, 2H), 8.25(s, 1H), 7.98 (d, *J* = 9.2 Hz, 1H), 7.78 (d, *J* = 8.5 Hz, 1H), 7.67 (d, *J* = 8.8 Hz, 2H), 7.55 (d, *J* = 8.4 Hz, 1H), 7.35 (t, *J* = 10.7 Hz, 2H), 7.33–7.16 (m, 2H), 6.44 (s, 1H), 4.04 (s, 3H), 3.58 (s, 3H), 2.83 (s, 3H), ^13^C-NMR (400 MHz, DMSO-*d*_6_) δ 154.08, 153.92, 152.32, 148.63, 139.21, 138.67, 138.05, 137.56, 129.21 × 2, 128.87 × 2, 128.55, 127.21, 121.98 × 2, 121.08, 118.98, 117.23, 115.18 × 2, 97.86, 54.77 × 2, 20.05. TOF MS ES^+^ (*m*/*z*): [M + H]^+^, calcd for C_25_H_23_ClN_4_O_5_S: 526.1078, found: 527.1156.

*4-(tert-Butyl)-N-((6-methoxy-4-((4-methoxyphenyl)amino)-2-methylquinolin-3-yl)carbamoyl)benzenesulfonamide* (**23b**). A pale white solid; Yield: 52.6%; m.p.: 291.2–292.7 °C; ^1^H-NMR (400 MHz, DMSO-*d*_6_) δ (ppm): 11.75 (d, *J* = 3.7 Hz, 1H), 7.89 (dd, *J* = 9.0, 6.4 Hz, 1H), 7.78 (d, *J* = 9.2 Hz, 2H), 7.62–7.53 (m, 2H), 7.47–7.40 (m, 2H), 7.33 (d, *J* = 8.2 Hz, 2H), 7.24 (s, 2H), 7.23–7.16 (m, 1H), 6.30 (s, 1H), 3.94 (s, 3H), 3.47 (s, 3H), 2.74 (d, *J* = 4.0 Hz, 3H), 1.55 (s, 9H), ^13^C-NMR (400 MHz, DMSO-*d*_6_) δ 156.28, 154.11, 153.94, 153.23, 148.73, 138.07, 137.94, 137.34, 130.28, 128.53 × 2, 127.00, 124.86 × 2, 121.9 × 2 121.30, 119.02, 118.12, 114.99 × 2, 98.72, 54.74 × 2, 37.18, 28.63 × 3, 20.74. TOF MS ES^+^ (*m*/*z*): [M + H]^+^, calcd for C_29_H_32_N_4_O_5_S: 548.2093, found: 549.2172.

*N-((4-((4-Bromophenyl)amino)-6-methoxy-2-methylquinolin-3-yl)carbamoyl)-4-fluorobenzenesulfonamide* (**23c**). A pale white solid; Yield: 40.3%; m.p.: 356.1–357.3 °C; ^1^H-NMR (400 MHz, DMSO-*d*_6_) δ (ppm): 11.83 (s, 1H), 7.93 (d, *J* = 8.4 Hz, 3H), 7.81 (d, *J* = 8.4 Hz, 2H), 7.32 (d, *J* = 9.4 Hz, 3H), 7.22 (dd, *J* = 9.2, 2.7 Hz, 2H), 7.07 (dd, *J* = 9.0, 2.5 Hz, 1H), 7.00 (d, *J* = 2.4 Hz, 1H), 6.29 (d, *J* = 2.5 Hz, 1H), 3.76 (s, 3H), 2.71 (s, 3H). ^13^C-NMR (400 MHz, DMSO-*d*_6_) δ 159.82, 155.81, 153.97, 152.07, 140.16, 139.41, 136.48, 132.45 × 2, 130.61, 130.44 × 2, 128.60, 127.75, 120.61, 118.52 × 2, 118.16, 114.79 × 2, 114.72, 98.94, 55.67, 20.15. TOF MS ES^+^ (*m*/*z*): [M + H]^+^, calcd for C_24_H_20_BrFN_4_O_4_S: 558.0373, found: 559.0451.

*N-((4-((3-Chloro-4-fluorophenyl)amino)-6-methoxy-2-methylquinolin-3-yl)carbamoyl)-4-fluorobenzenesulfonamide* (**23d**). A pale white solid; Yield: 39.5%; m.p.: 285.3–286.1 °C; ^1^H-NMR (400 MHz, DMSO-*d*_6_) δ (ppm): 11.87 (s, 1H), 8.34 (s, 1H), 7.81 (d, *J* = 5.8 Hz, 1H), 7.77–7.59 (m, 2H), 7.50 (d, *J* = 8.5 Hz, 2H), 7.40(s, 1H) 7.18 (q, *J* = 9.1 Hz, 2H), 7.01 (dd, *J* = 18.2, 8.8 Hz, 1H), 6.96–6.88 (m, 1H), 6.04 (d, *J* = 2.2 Hz, 1H), 3.23 (s, 3H), 2.26 (s, 3H), ^13^C-NMR (400 MHz, DMSO-*d*_6_) δ 156.33, 154.04, 140.82, 140.04, 135.10, 133.00, 131.80, 131.42, 129.37 × 2, 128.67, 128.23, 121.34, 120.85, 118.87 × 2, 118.56, 117.05, 115.91 × 2, 115.01, 99.35, 54.95, 20.73. TOF MS ES^+^ (*m*/*z*): [M + H]^+^, calcd for C_24_H_19_ClF_2_N_4_O_4_S: 532.0784, found: 533.0862.

^1^H-NMR spectra of all the target compounds, ^13^C-NMR spectra of representative target compounds (**19a**, **19c**, **20c**, **21b**, **21c**, **21d** and **22b**) and TOF-MS spectra of representative target compounds (**19a**, **19c**, **22b** and **23b**) can be seen in the Supplementary Materials.

### 3.3. PI3Kα Kinase Assay

The potent compounds **19a** and **23b** were tested for their activities against PI3Kα enzyme using Kinase-Glo Luminescent Kinase Assay (Promega, Madison, WI, USA), with NVPBEZ-235 and PI101 as positive control. The kinase reaction was done in a 384-well black plate. Each well was loaded with 50 µL of test items (in 90% DMSO) and 5 µL reaction buffer containing 10 µg/mL PI substrate (l-α-phosphatidylinositol); Avanti Polar Lipids (Avanti Polar Lipids, Inc., Alabaster, AL, USA); prepared in 3% octyl-glucoside) and the PI3Ka protein 10 nM was then added to it. The reaction was started by the addition of 5 µL of 1 µM ATP prepared in the reaction buffer (50 mM HEPES pH 7.5, 1 mM EGTA, 3 mM MnCl_2_, 10 mM MgCl_2_, 2 mM DTT and 0.01% Tween-20) and was incubated for 60 min. It was terminated by the addition of 10 µL Kinase-Glo buffer. The plates were then read in Synergy 2 readers (BioTek, Winooski, VT, USA) for luminescence detection. The assay was repeated two times and the results expressed as IC_50_ (inhibitory concentration 50%) were the averages of two determinations.

### 3.4. mTOR Kinase Assay

The potent compounds **19a** and **23b** were tested for their activities against mTOR enzyme using Kinase-Glo Luminescent Kinase Assay (Promega, Madison, WI, USA), with NVPBEZ-235 and PI101 as positive control. The kinase reaction was done in a 384-well black plate. Each well was loaded with 50 µL of test items (in 90% DMSO) and 5 µL reaction buffer containing 10 µg/mL PI substrate (l-α-phosphatidylinositol); Avanti Polar Lipids (Avanti Polar Lipids, Inc., Alabaster, AL, USA); prepared in 3% octyl-glucoside) and the mTOR protein 2.5 nM was then added to it. The reaction was started by the addition of 5 µL of 10 µM ATP prepared in the reaction buffer (50 mM HEPES pH 7.5, 1 mM EGTA, 3 mM MnCl_2_, 10 mM MgCl_2_, 2 mM DTT and 0.01% Tween-20) and was incubated for 60 min. It was terminated by the addition of 10 µL Kinase-Glo buffer. The plates were then read in Synergy 2 readers (BioTek, Winooski, VT, USA) for luminescence detection. The assay was repeated two times and the results expressed as IC_50_ (inhibitory concentration 50%) were the averages of two determinations.

### 3.5. Cytotoxicity Assay In Vitro

The in vitro cytotoxic activities of all compounds **19a**–**d**, **20a**–**d**, **21a**–**d**, **22a**–**d** and **23a**–**d** were evaluated with HepG-2, A549, PC-3 and MCF-7 cell lines by the standard MTT assay, with NVPBEZ235 and Sorafenib as positive control. The cancer cell lines were cultured in minimum essential medium (MEM) supplement with 10% fetal bovine serum (FBS). Approximately 4 × 10^3^ cells, suspended in MEM medium, were plated onto each well of a 96-well plate and incubated in 5% CO_2_ at 37 °C for 24 h. The test compounds at indicated final concentrations were added to the culture medium and the cell cultures were continued for 72 h. Fresh MTT was added to each well at a terminal concentration of 5 µg/mL and incubated with cells at 37 °C for 4 h. The formazan crystals were dissolved in 100 µL DMSO each well, and the absorbency at 492 nm (for absorbance of MTT formalin) and 630 nm (for the reference wavelength) was measured with an ELISA reader. All of the compounds were tested three times in each of the cell lines. The results were expressed as inhibition rates or IC_50_ (half-maximal inhibitory concentration) were the averages of two determinations and calculated by using the Bacus Laboratories Inc. Slide Scanner (Bliss) software (the Bacus Laboratories Inc. Slide Scanner (BLISS) system, Lombard, II, USA).

### 3.6. Docking Studies

Molecular docking simulation studies were carried out by the AutoDock 4.2 software (The Scripps Research Institute, USA). The docking tutorial we used and the detailed AutoDock basic operational methods can be found at: http://autodock.scripps.edu/faqs-help/tutorial. The protein preparation process of flexible docking mainly includes fixing the exact residues, adding hydrogen atoms, removing irrelevant water molecules, adding charges, etc. The potent compounds were selected as ligand examples, and the structures of PI3Kγ (PDB code: 3L08, http://www.pdb.org/) were selected as the docking models. Only the best-scoring ligand–protein complexes were used for the binding site analyses. All the docking results were processed and modified in Open-Source PyMOL 1.8. x software (https://pymol.org).

## 4. Conclusions

In summary, five series of phenylsulfonylurea derivatives, **19a**–**d**, **20a**–**d**, **21a**–**d**, **22a**–**d** and **23a**–**d**, bearing 4-phenylaminoquinoline scaffolds were designed and synthesized. In addition, we evaluated them for antitumor activity against four cancer cell lines and both PI3Kα kinase and mTOR kinase activity (only compounds **19a** and **23b**) in vitro. The pharmacological results indicated that most of the compounds showed moderate cytotoxicity activity against the four cancer cell lines. In particular, the cellular activity of the potent compounds **19a** and **23b** against MCF-7 cell was equal to the positive control Soraffenib, with the IC_50_ value of 2.88 ± 0.58 and 6.55 ± 0.81, respectively. Structure–activity relationships (SARs) indicated that the introduction of the methoxyl scaffold into 4-anilinoquinoline scaffold was more favorable than the introduction of halogen to the cellular activity. In general, the methoxy substitution and halogen substitution at 6-position of the quinoline ring played no significant impact on the activity. The para-methoxyl substitution of 4-anilino moiety and para-halogen substitution of phenylsulfonylurea have different impacts on different series of compounds. Moreover, the oxygen atom of phenylsulfonylurea structure can form a hydrogen bond with the residue LYS833 of PI3Kγ protein. Further studies will be carried out in the near future.

## Figures and Tables

**Table 1:**
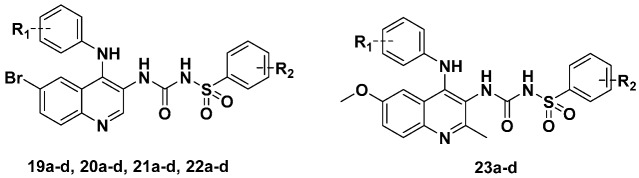
Structures and cytotoxicity of compounds **19a**–**d**, **20a**–**d**, **21a**–**d**, **22a**–**d** and **23a**–**d**.

Compound	R_1_	R_2_	IC_50_ (µM) ^a^			
			HepG-2	A549	PC-3	MCF-7
**19a**	pivalonitrile	-F	33.28 ± 1.52	38.52 ± 1.04	66.62 ± 1.28	3.88 ± 0.58
**19b**	pivalonitrile	-Cl	30.22 ± 1.45	>100	34.31 ± 1.32	15.10 ± 1.1
**19c**	pivalonitrile	-CH3	31.25 ± 1.50	28.96 ± 1.31	40.59 ± 1.11	3.96 ± 0.59
**19d**	pivalonitrile	Tert-Butyl	>100	30.58 ± 1.45	68.25 ± 1.32	9.95 ± 0.99
**20a**	-OCH3	-F	20.34 ± 1.04	26.34 ± 1.13	>100	7.65 ± 0.88
**20b**	-OCH3	-Cl	22.56 ± 1.12	30.22 ± 1.23	>100	10.65 ± 0.97
**20c**	-OCH3	-CH3	26.83 ± 1.22	23.35 ± 1.12	>100	4.94 ± 0.64
**20d**	-OCH3	Tert-Butyl	33.24 ± 1.26	32.12 ± 1.18	>100	10.46 ± 0.98
**21a**	-Br	-F	45.68 ± 1.66	58.98 ± 1.76	>100	37.59 ± 1.57
**21b**	-Br	-Cl	11.13 ± 1.04	71.11 ± 1.85	>100	13.78 ± 1.14
**21c**	-Br	-CH3	31.05 ± 1.49	22.74 ± 1.35	>100	16.63 ± 1.22
**21d**	-Br	Tert-Butyl	16.97 ± 1.22	16.16 ± 1.20	>100	52.67 ± 1.72
**22a**	-3-Cl-4-F	-F	19.63 ± 0.18	12.02 ± 0.91	52.52 ± 1.03	22.83 ± 1.35
**22b**	-3-Cl-4-F	-Cl	6.443 ± 0.89	>100	17.72 ± 0.95	6.84 ± 0.83
**22c**	-3-Cl-4-F	-CH3	11.274 ± 0.84	>100	16.74 ± 0.88	16.81 ± 1.22
**22d**	-3-Cl-4-F	Tert-Butyl	28.43 ± 0.89	22.40 ± 1.21	29.27 ± 2.23	46.30 ± 1.67
**23a**	-OCH3	-Cl	15.98 ± 1.10	39.76 ± 1.59	>100	6.30 ± 0.79
**23b**	-OCH3	Tert-Butyl	2.71 ± 0.43	7.47 ± 0.87	>100	6.55 ± 0.81
**23c**	-Br	-F	32.12 ± 1.12	12.96 ± 0.84	39.76 ± 1.25	25.44 ± 1.43
**23d**	-3-Cl-4-F	-F	6.08 ± 1.04	38.14 ± 0.96	57.85 ± 0.99	57.03 ± 1.75
**NVP-BEZ235 ^b^**	-	-	0.54 ± 0.83	0.36 ± 0.86	0.20 ± 0.11	0.14 ± 0.10
**Sorafenib ^b^**	-	-	3.97 ± 0.13	6.53 ± 0.23	3.03 ± 0.11	4.21 ± 0.15

^a^ The values are an average of two separate determinations; ^b^ used as a positive control.

**Table 2:** Enzymatic activities of compounds **19a** and **23b** against PI3Kα and mTOR (IC_50_, µM).

Compound	IC_50_(µM)	
	PI3Kα	mTOR
**19a**	0.72	2.62
**23b**	>10	>10
**NVP-BEZ235**	0.004 ± 0.002	0.006 ± 0.003
**PI101**	0.011 ± 0.002	0.019 ± 0.002

PI3Kα: Phosphatidylinositol-3-kinase alpha subunit; mTOR: Mammalian target of rapamycin.

## Supplementary Materials

The Supplementary Materials are available online at http://www.mdpi.com/1420-3049/23/7/1553/s1.
