# Supplementary material for: Design, Synthesis and Biological Evaluation of Novel Phenylsulfonylurea Derivatives as PI3K/mTOR Dual Inhibitors
Source: Molecules. 2018 Jun 27;23(7):1553. doi: 10.3390/molecules23071553 (PMC6099940; doi:10.3390/molecules23071553)
Supplement: Supplementary file 1 [file molecules-23-01553-s001.pdf]

Supplementary Materials: Design, Synthesis and Biological Evaluation of Novel Phenylsulfonylurea Derivatives as PI3K/mTOR Dual Inhibitors

Bingbing Zhao, Fei Lei, Caolin Wang, Binliang Zhang, Zunhua Yang, Wei Li, Wufu Zhu, and Shan Xu

Content

^{1}H-NMR spectra of compound **19a-d, 20a-d, 21a-d, 22a-d** and **23a-d.**

^{13}C-NMR spectra of compound **19a, 19c, 20c, 20b, 21c, 21d** and **22b.**

TOF MS spectra of compound **19a, 19c, 22b** and **23b.**

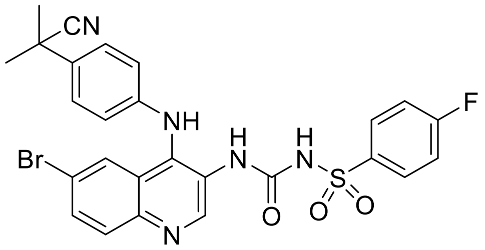

Compound 19a

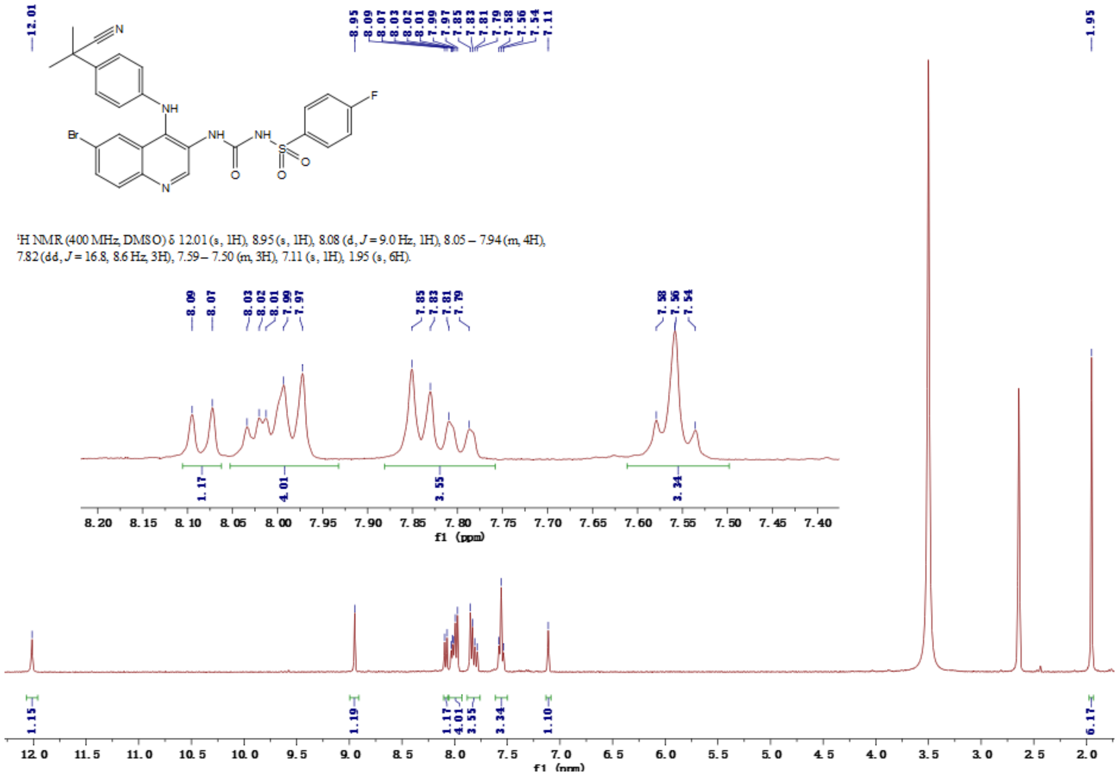

Figure S1. ^{1}H-NMR of compound 19a.

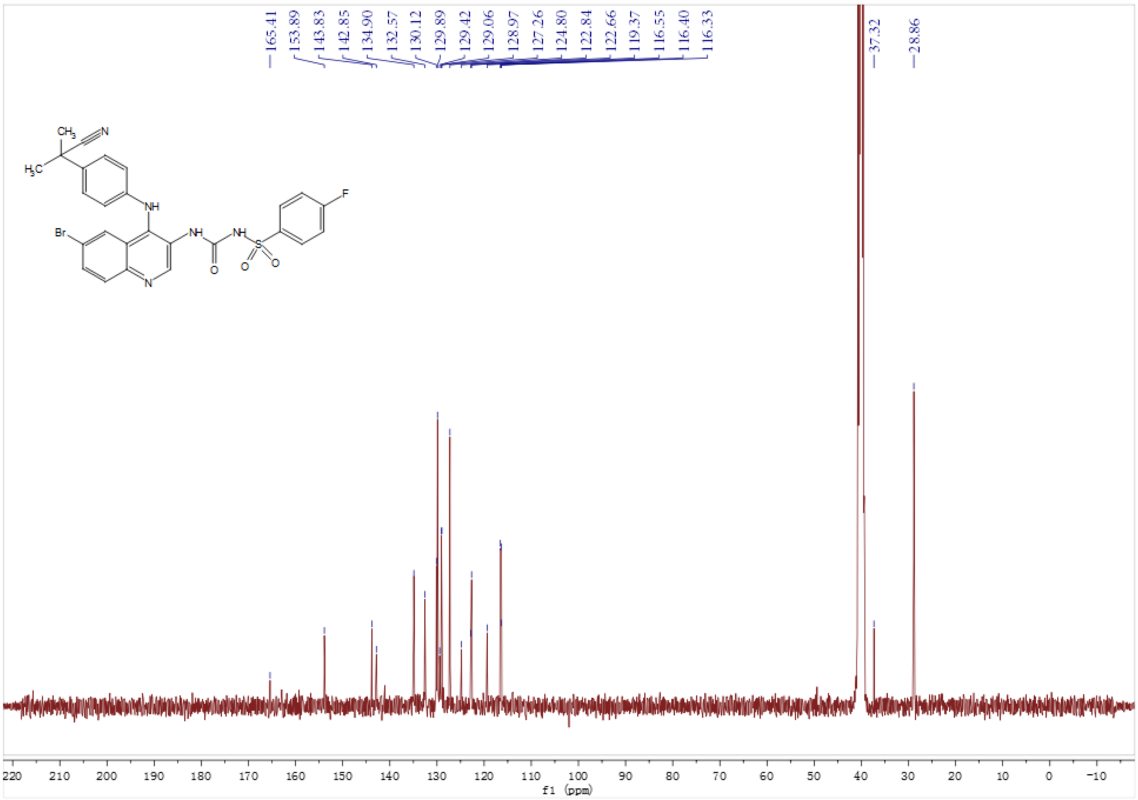

Figure S2. ^{13}C-NMR of compound 19a.

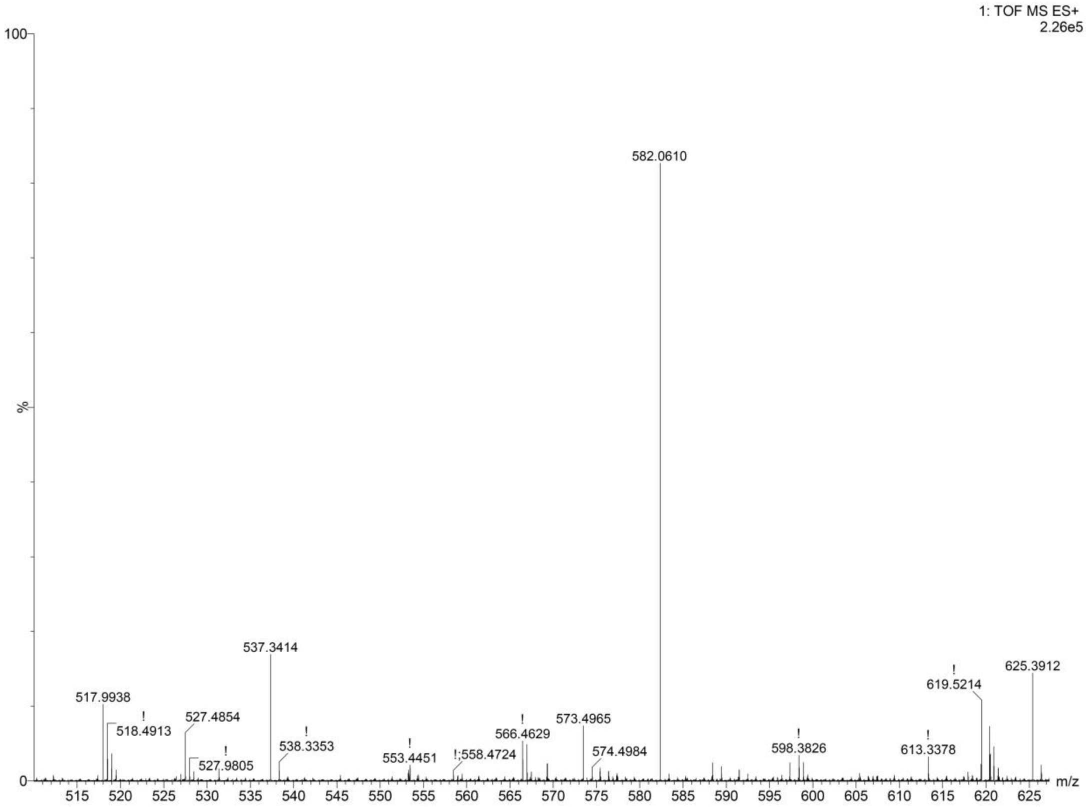

Figure S3. TOF MS of compound 19a.

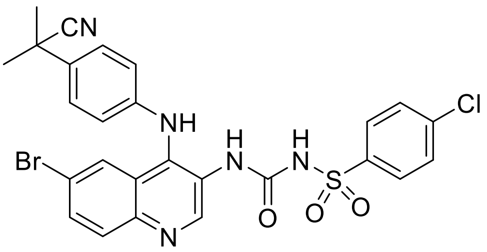

Compound 19b

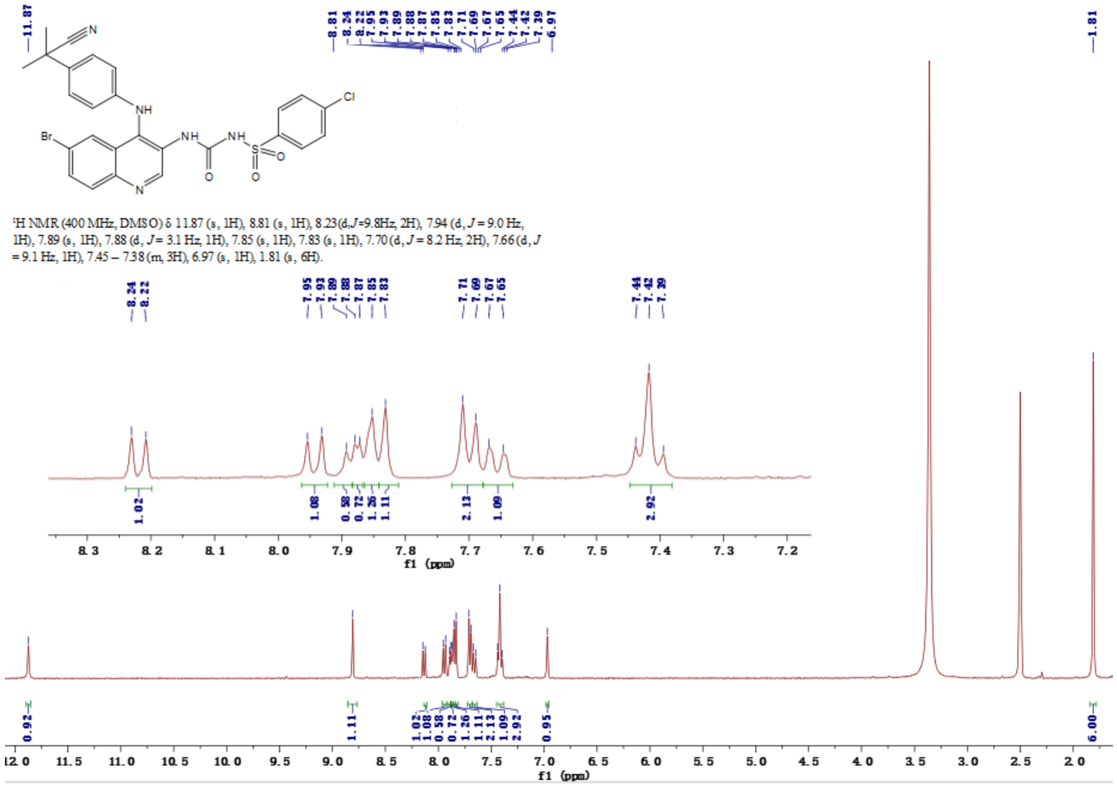

Figure S4. ^{1}H-NMR of compound 19b.

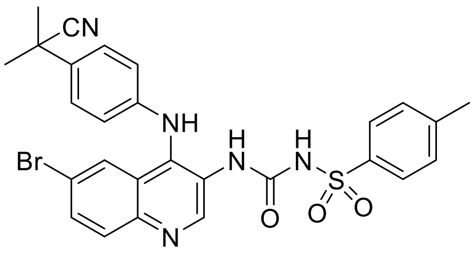

Compound 19c

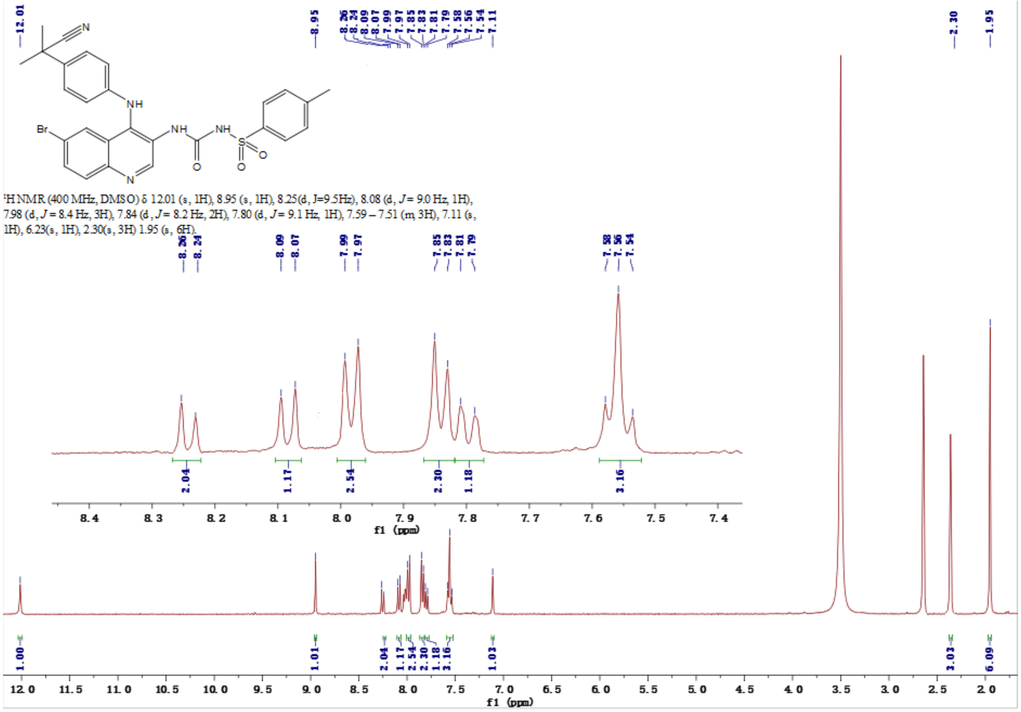

Figure S5. ^{1}H-NMR of compound 19c.

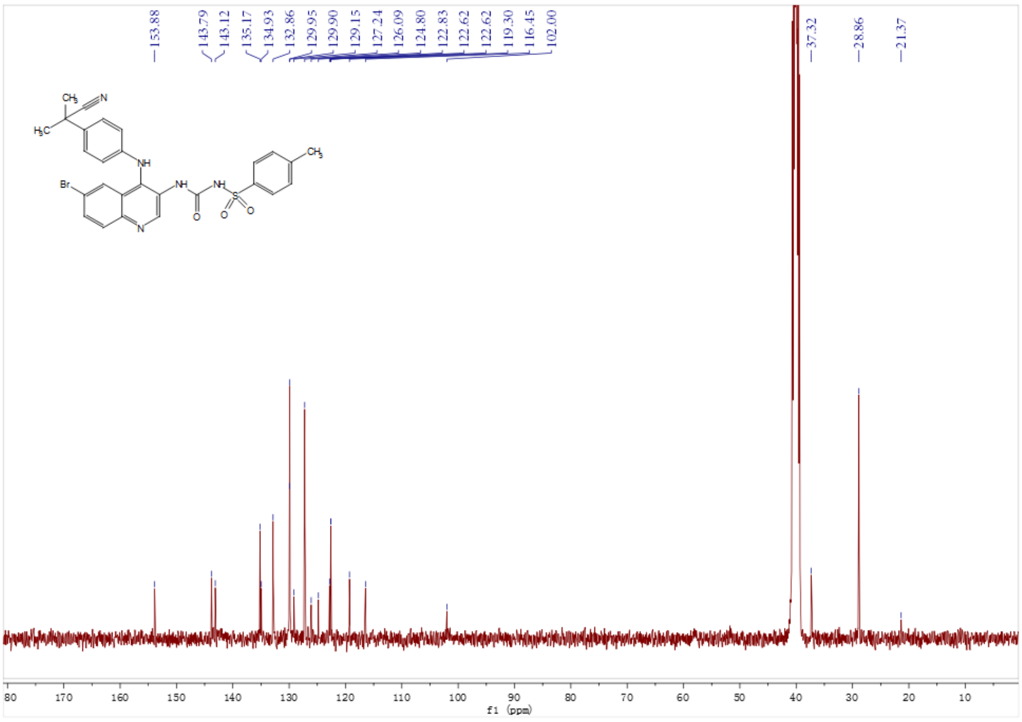

Figure S6. ^{13}C-NMR of compound 19c.

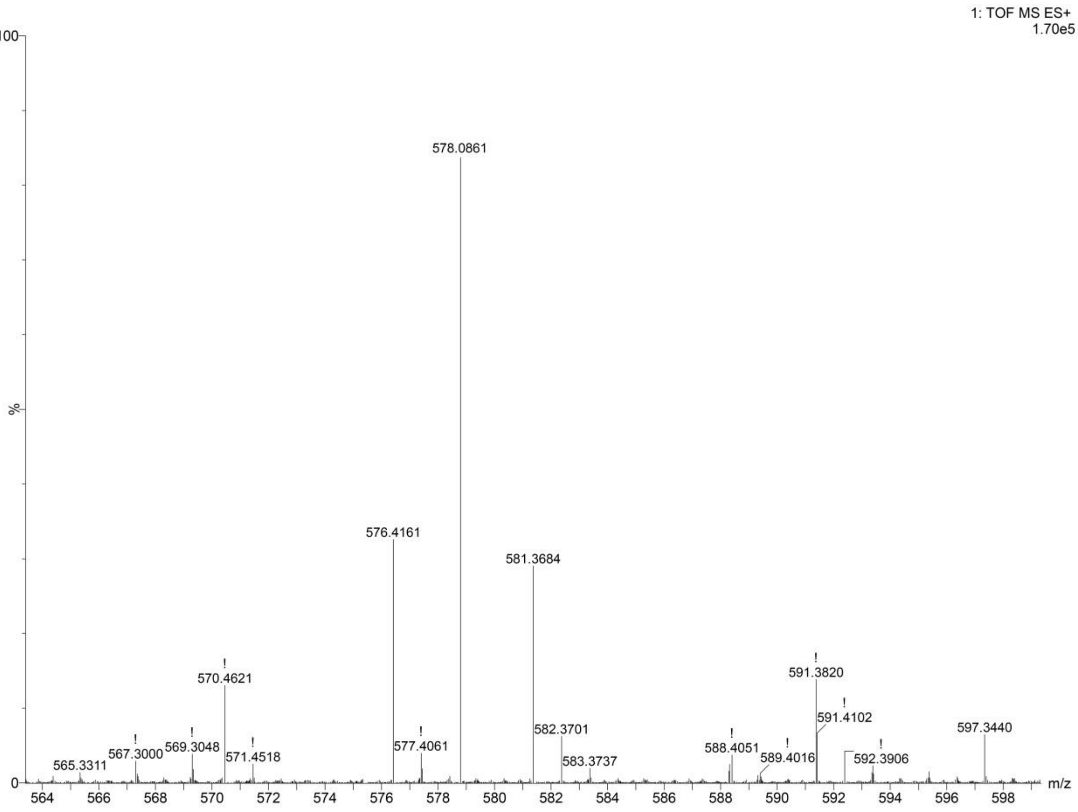

Figure S7. TOF MS of compound 19c.

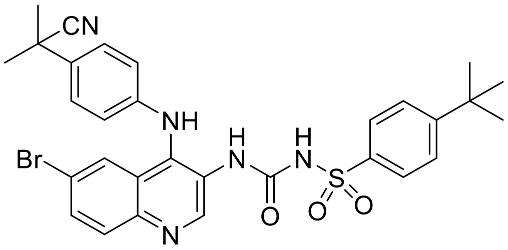

Compound 19d

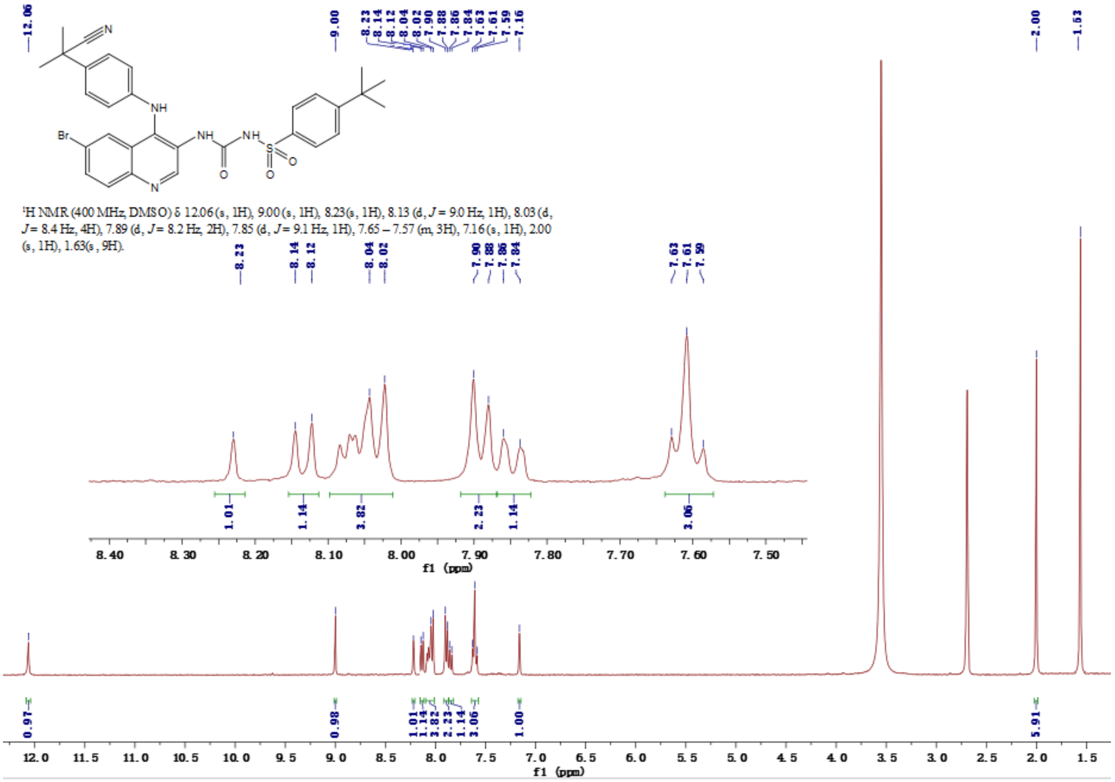

Figure S8. ^{1}H-NMR of compound 19d.

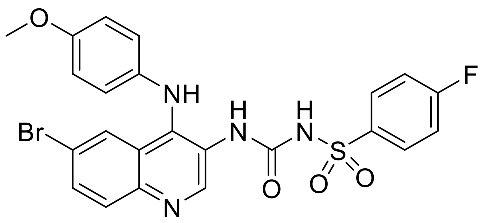

Compound 20a

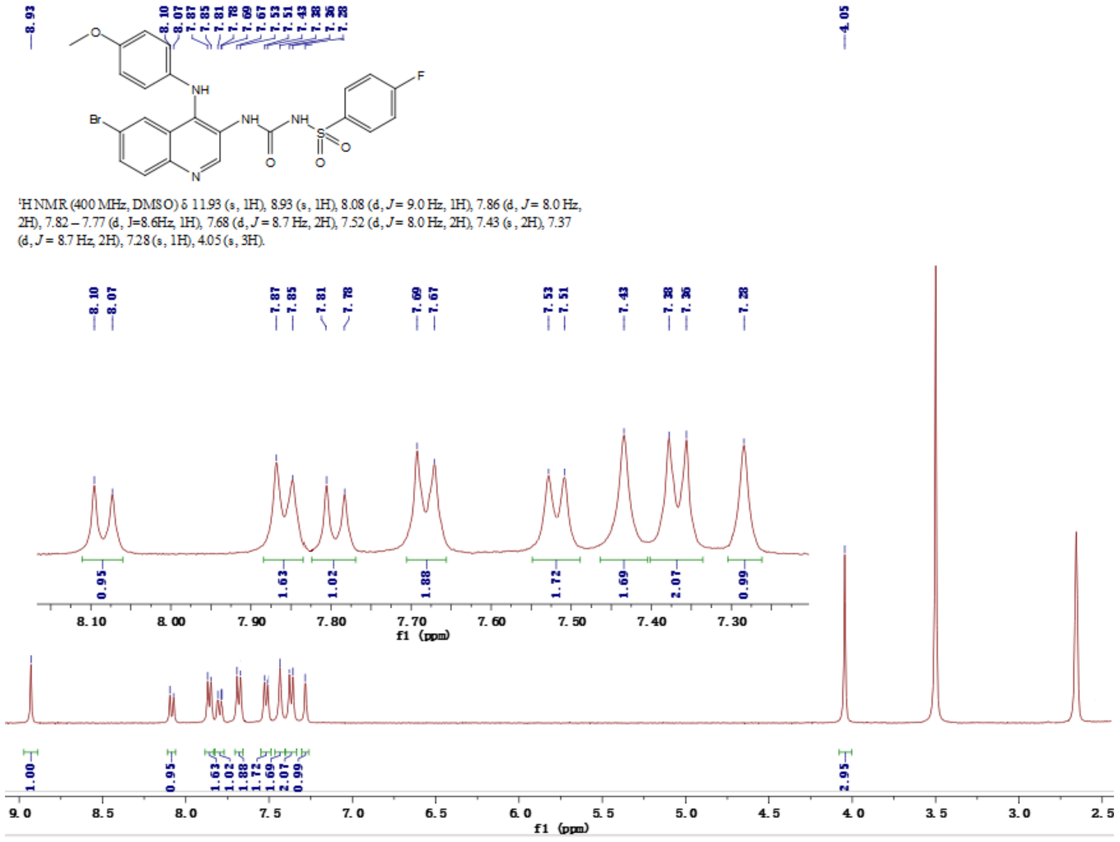

Figure S9. ^{1}H-NMR of compound 20a.

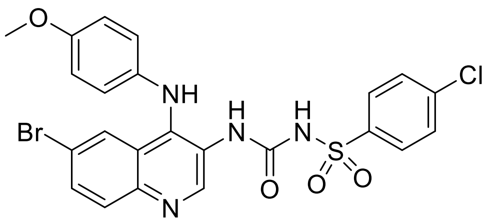

Compound 20b

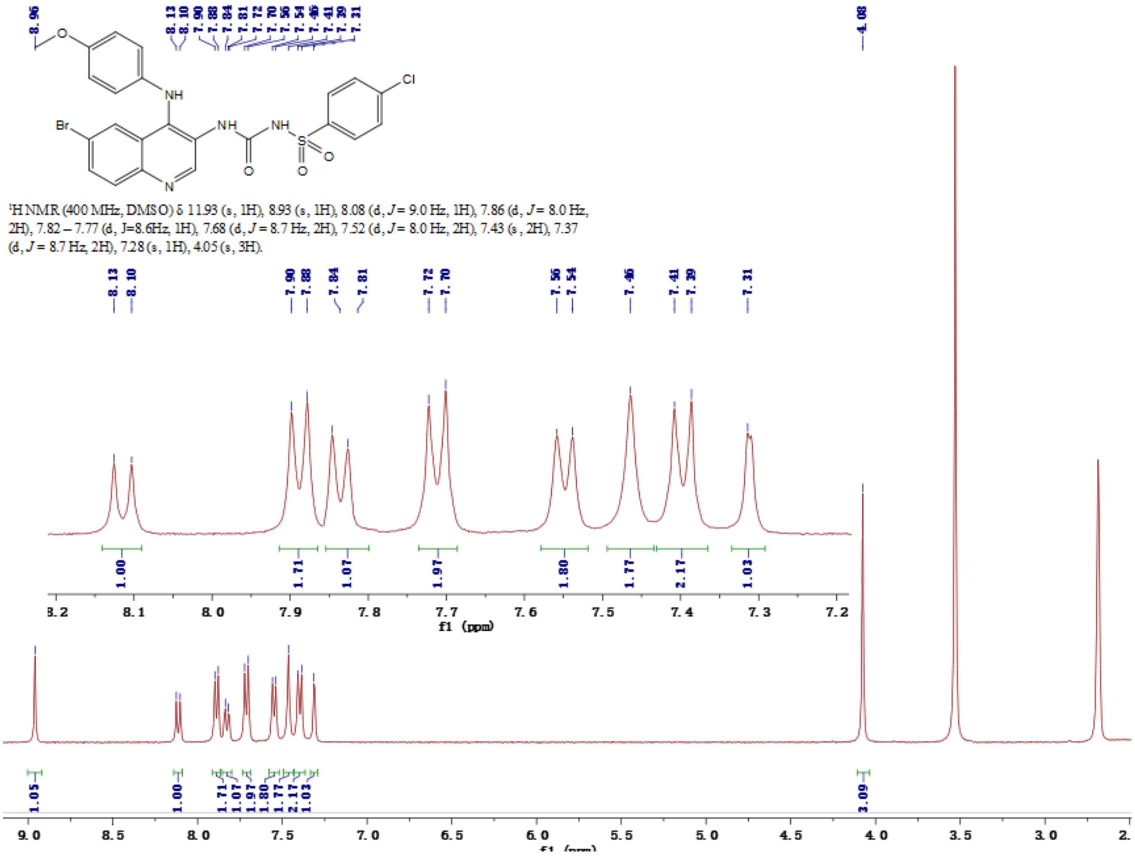

Figure S10. ^{1}H-NMR of compound 20b.

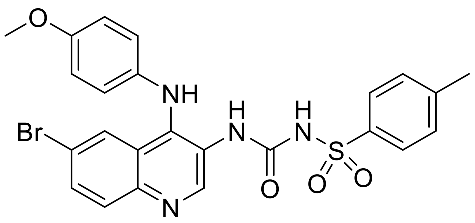

Compound 20c

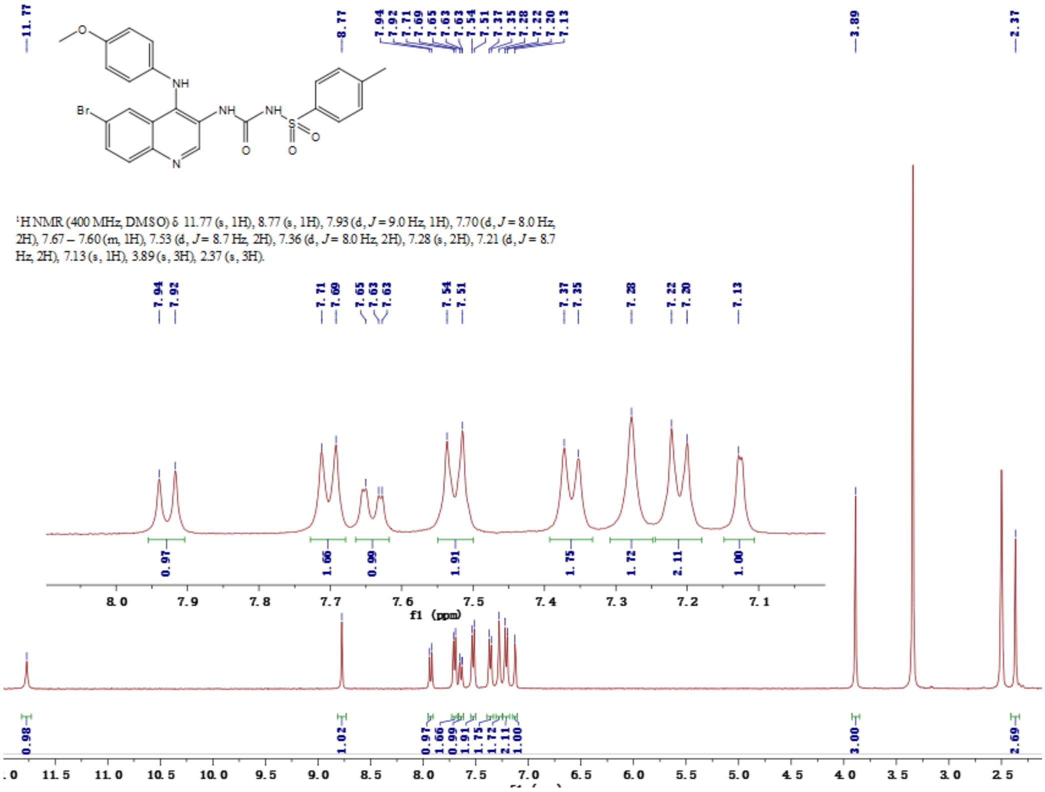

Figure S11. ^{1}H-NMR of compound 20c.

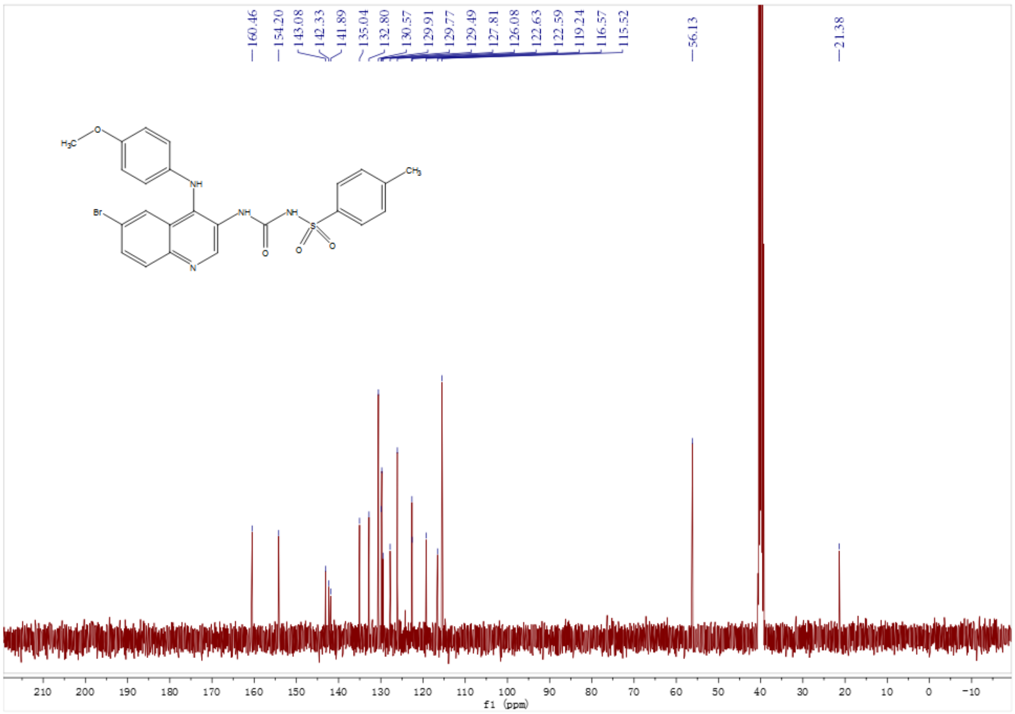

Figure S12. ^{13}C-NMR of compound 20c.

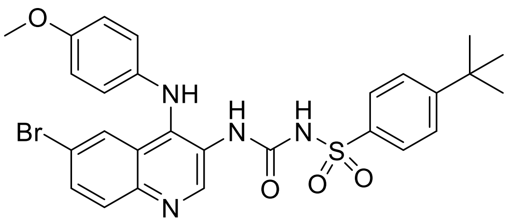

Compound 20d

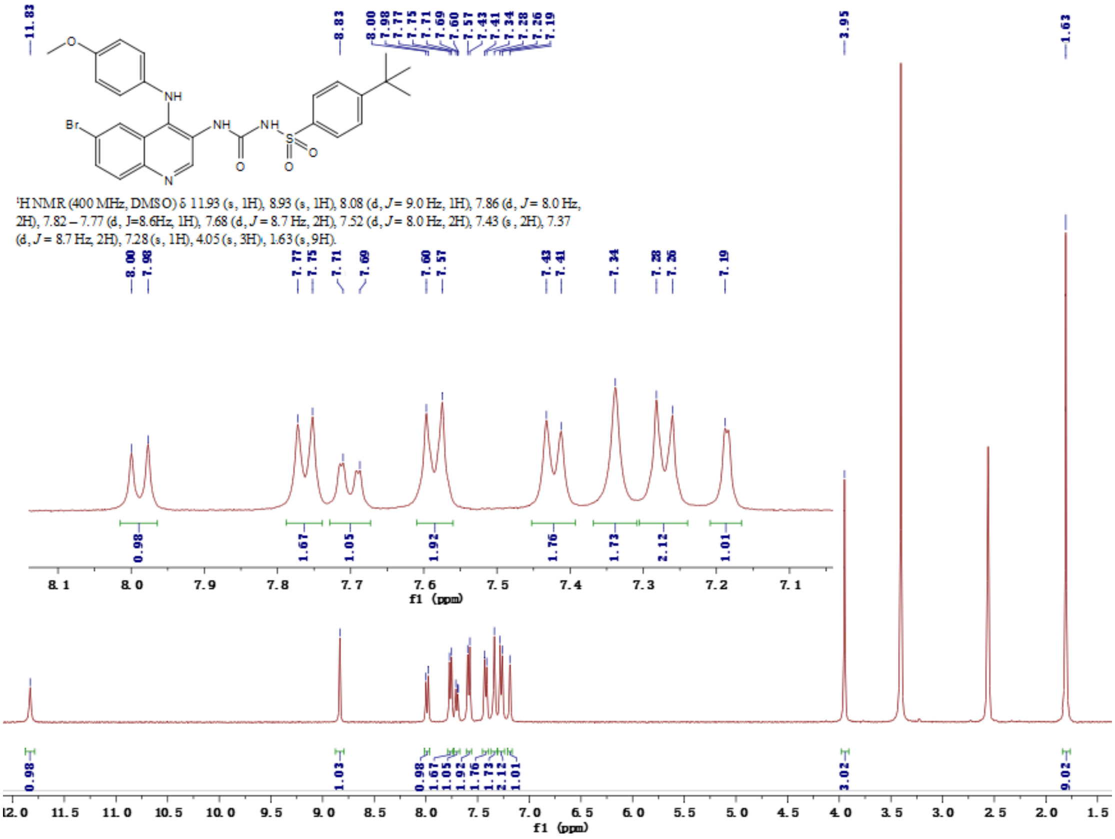

Figure S13. ^{1}H-NMR of compound 20d.

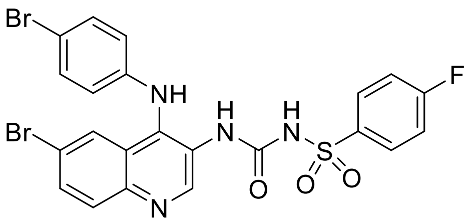

Compound 21a

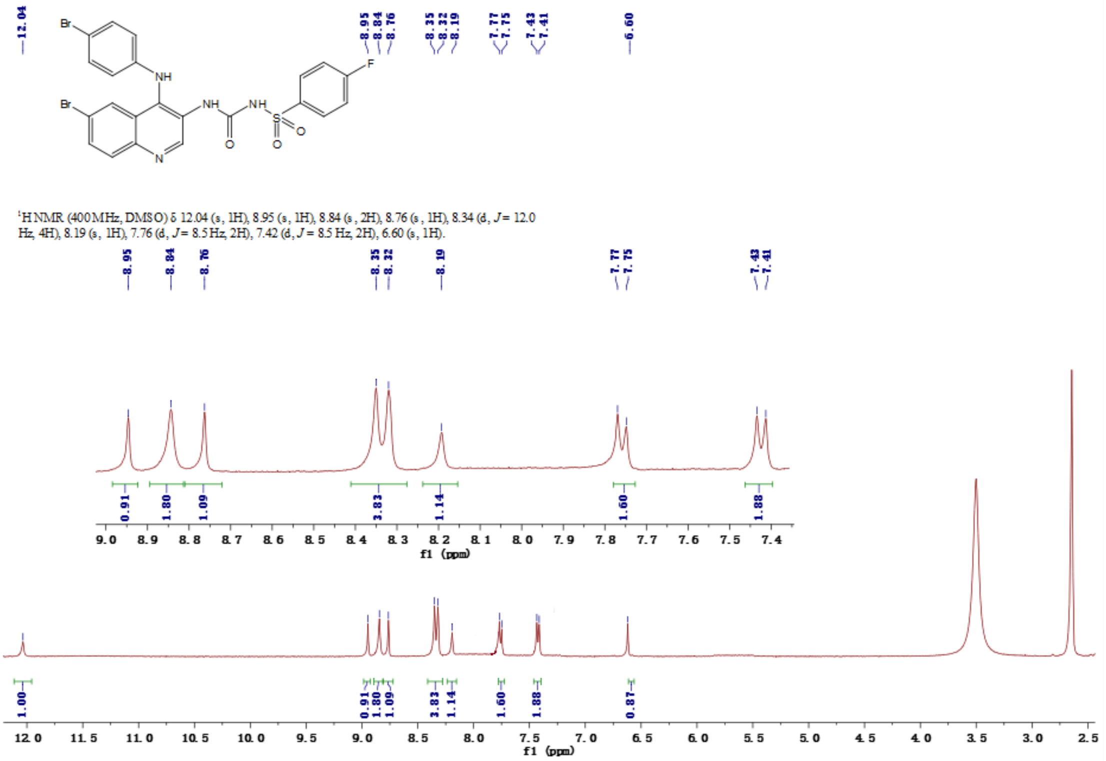

Figure S14. ^{1}H-NMR of compound 21a.

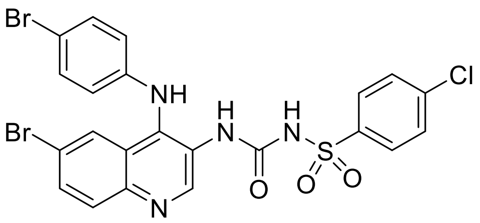

Compound 21b

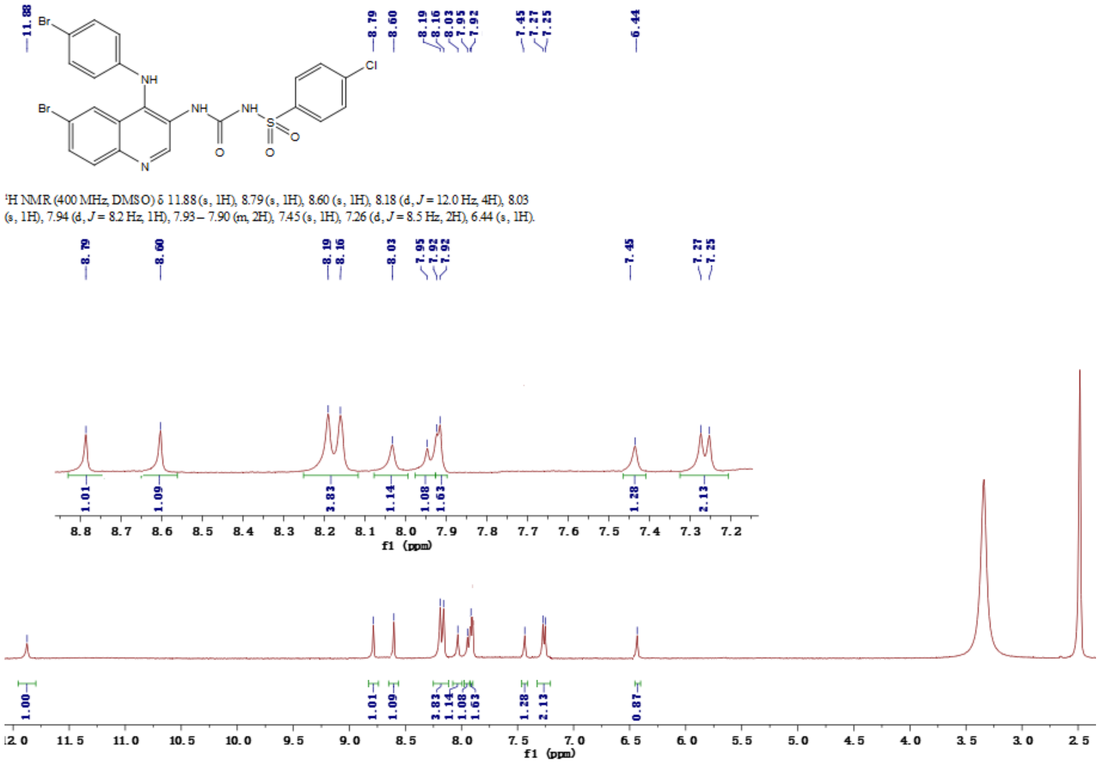

Figure S15. ^{1}H-NMR of compound 21b.

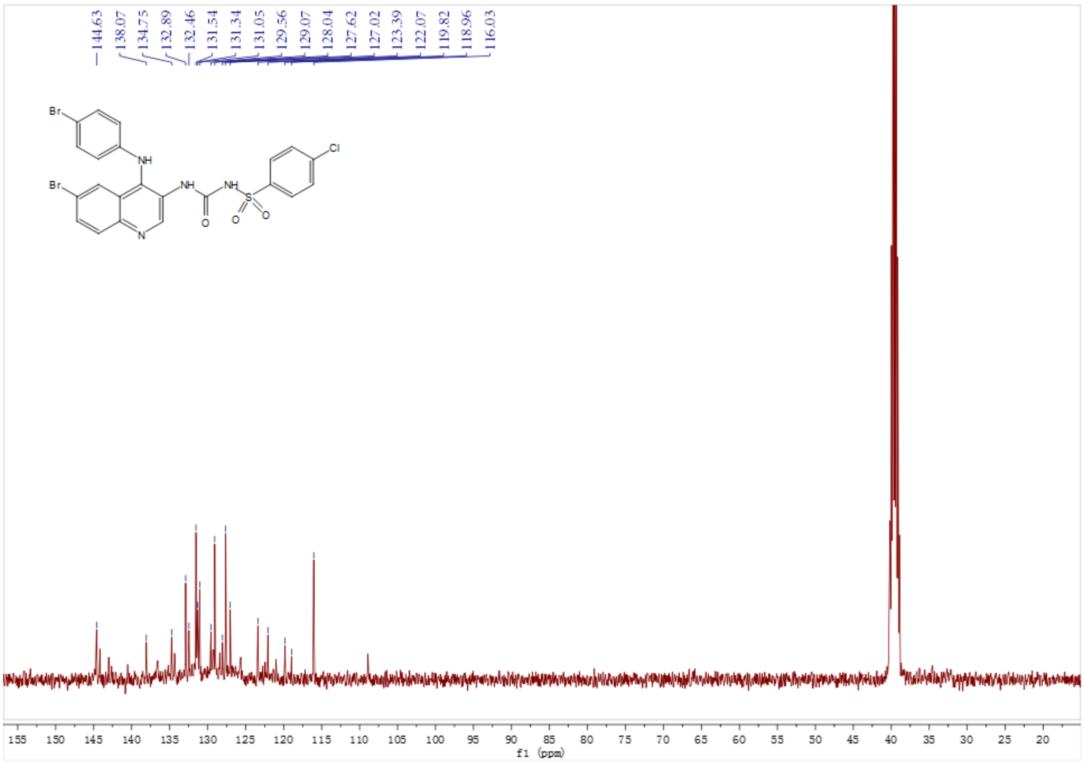

Figure S16. ^{13}C-NMR of compound 21b.

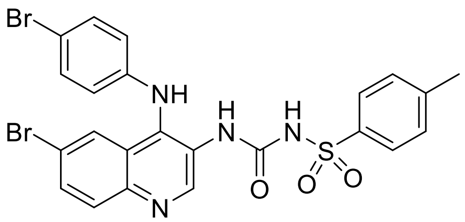

Compound 21c

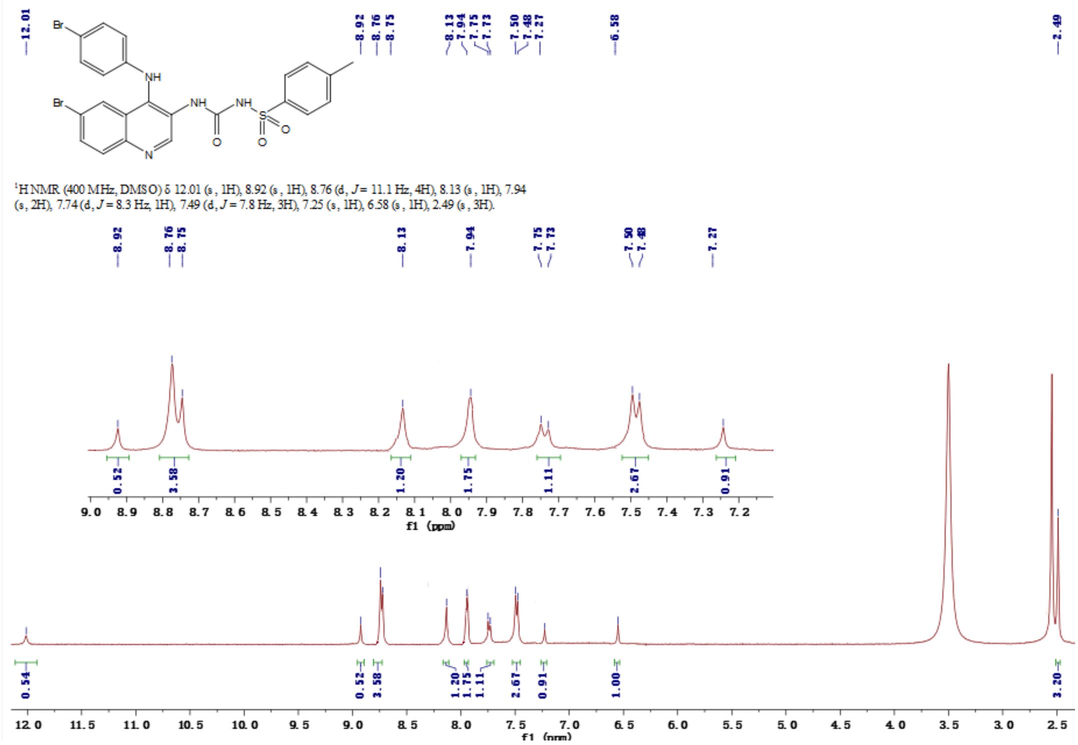

Figure S17. ¹H-NMR of compound 21c.

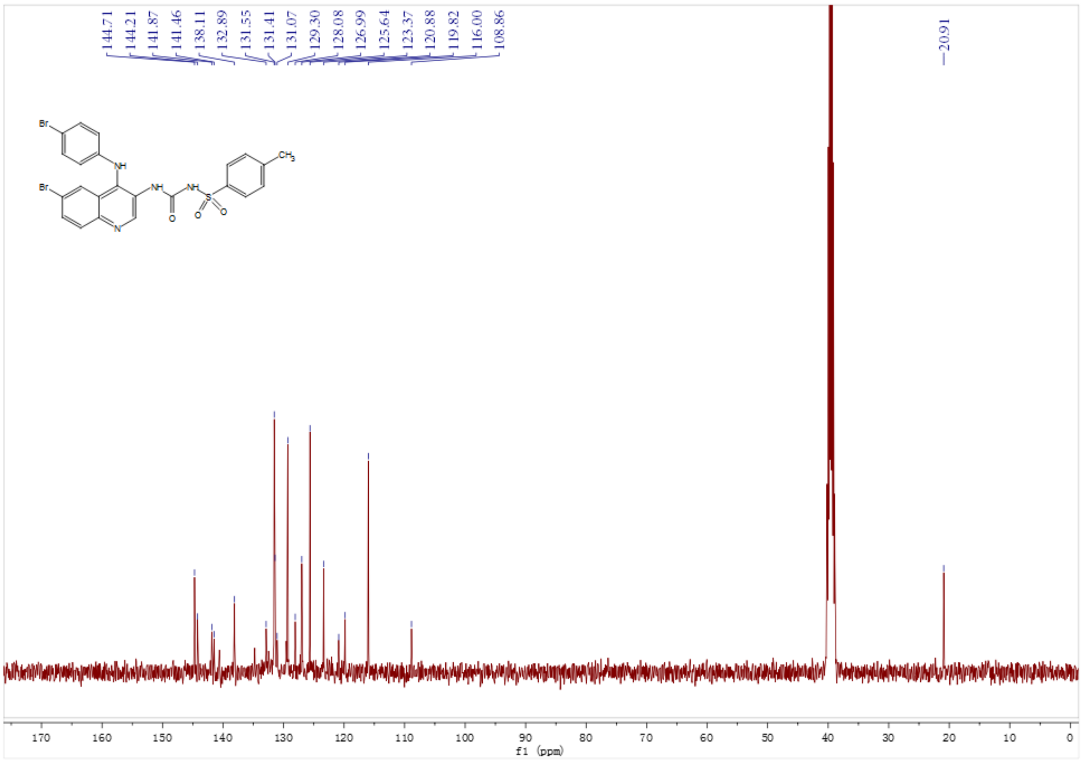

Figure S18. ¹³C-NMR of compound 21c.

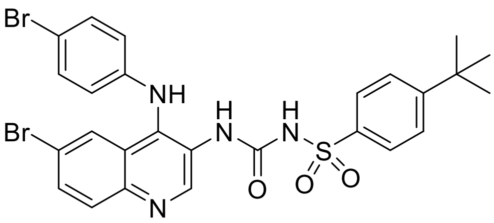

Compound 21d

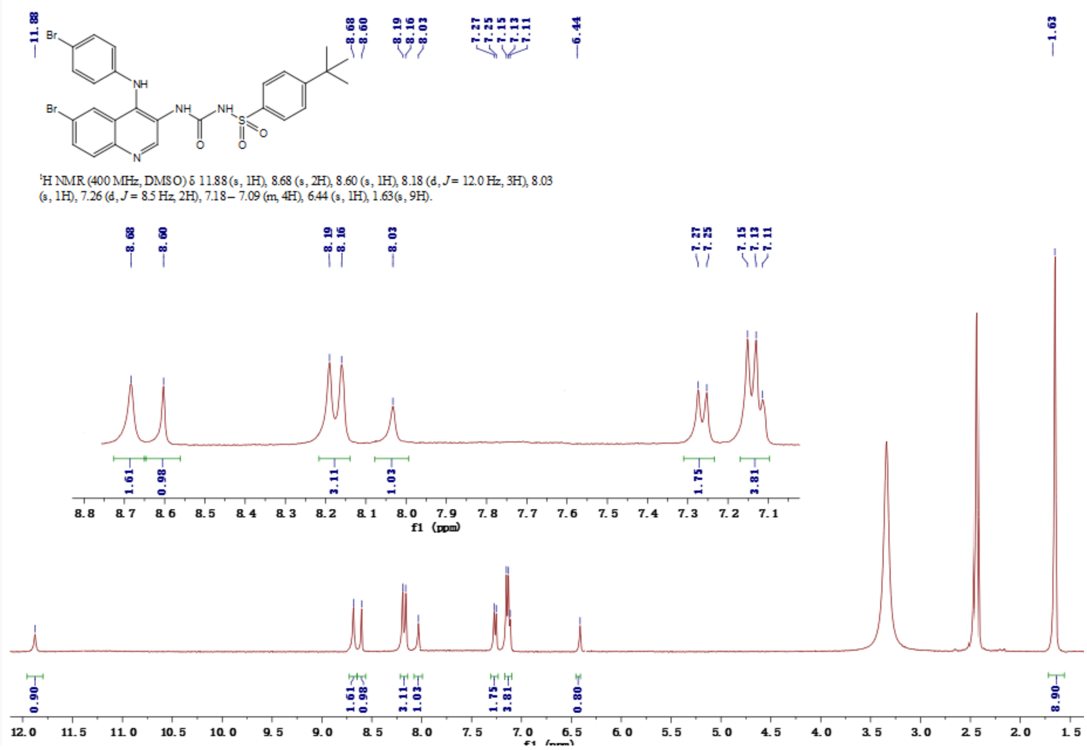

Figure S19. ^{1}H-NMR of compound 21d.

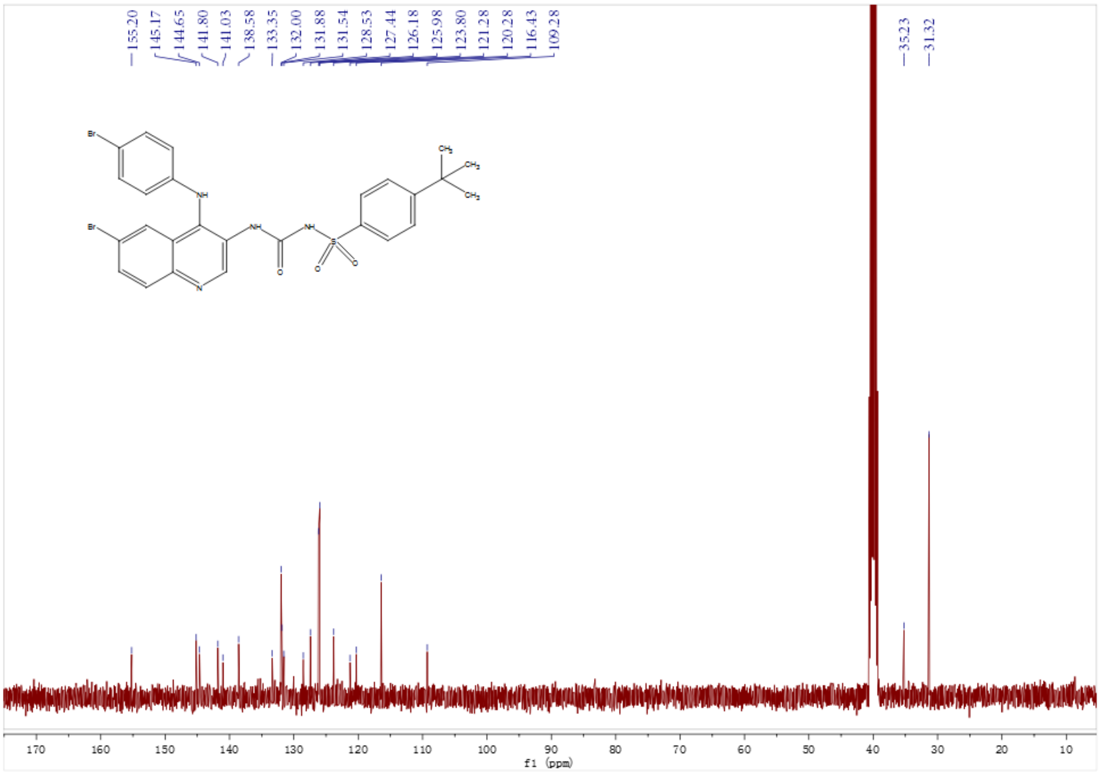

Figure S20. ^{13}C-NMR of compound 21d.

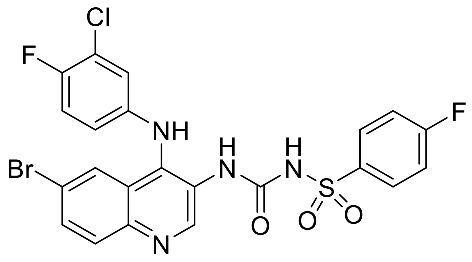

Compound 22a

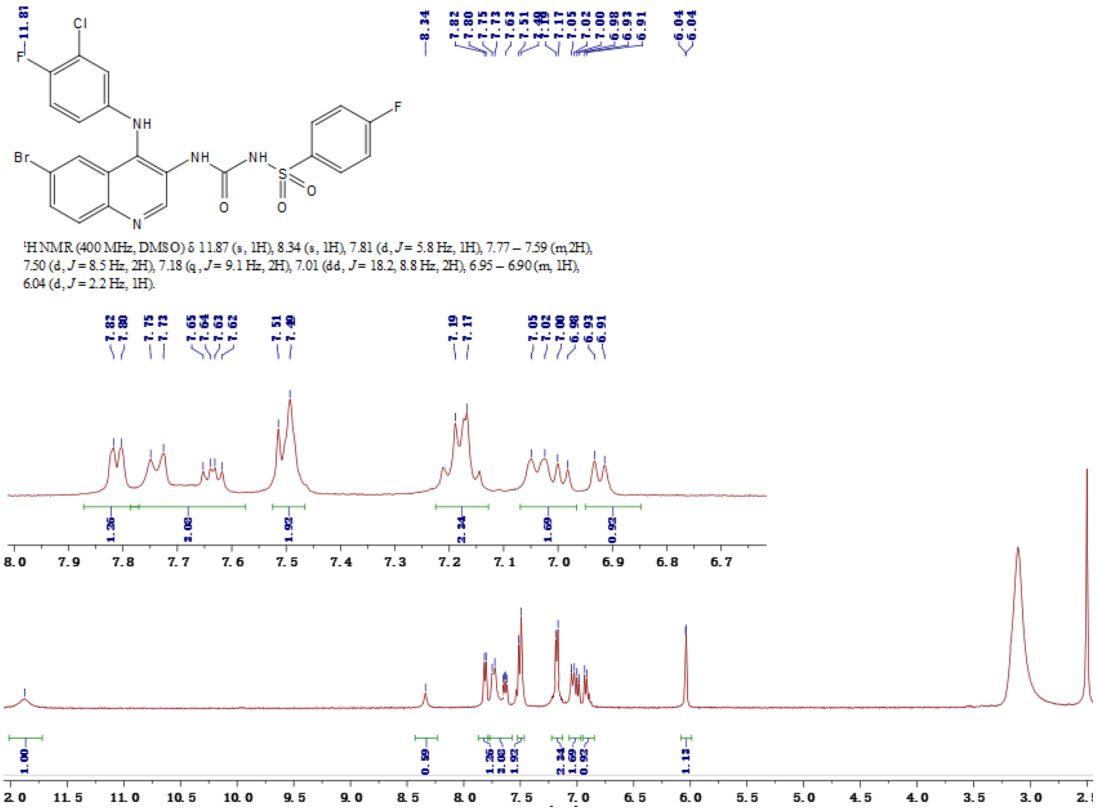

Figure S21. ^{1}H-NMR of compound 22a.

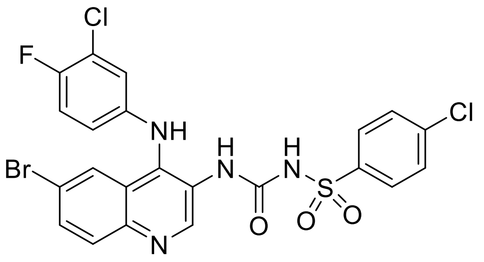

Compound 22b

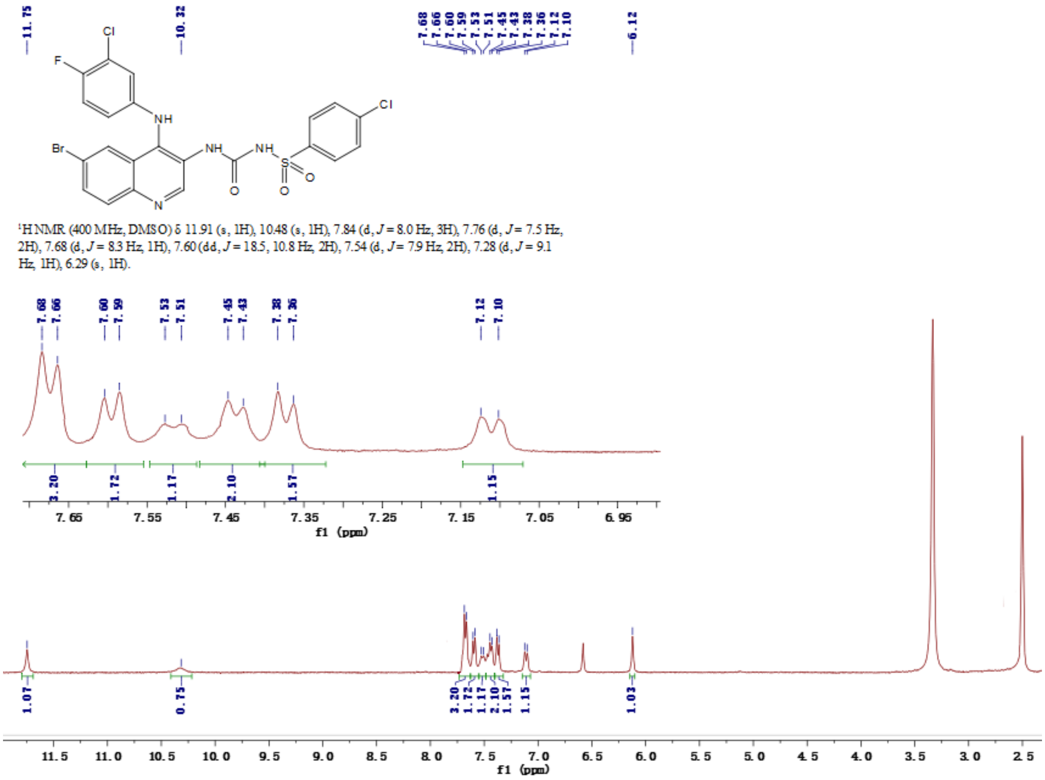

Figure S22. ^{1}H-NMR of compound 22b.

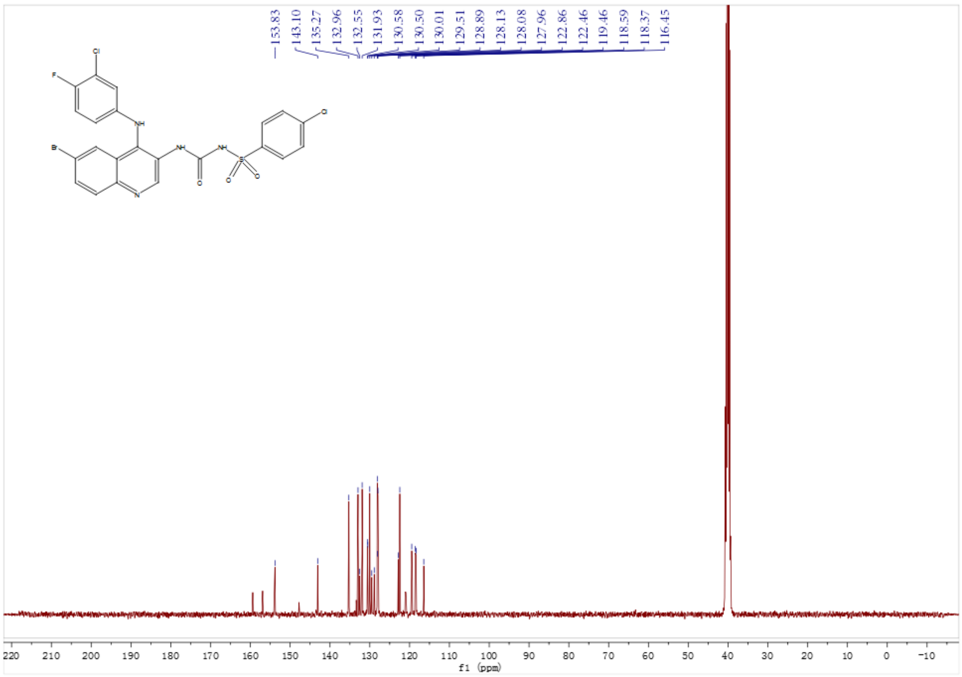

Figure S23. ^{13}C-NMR of compound 22b.

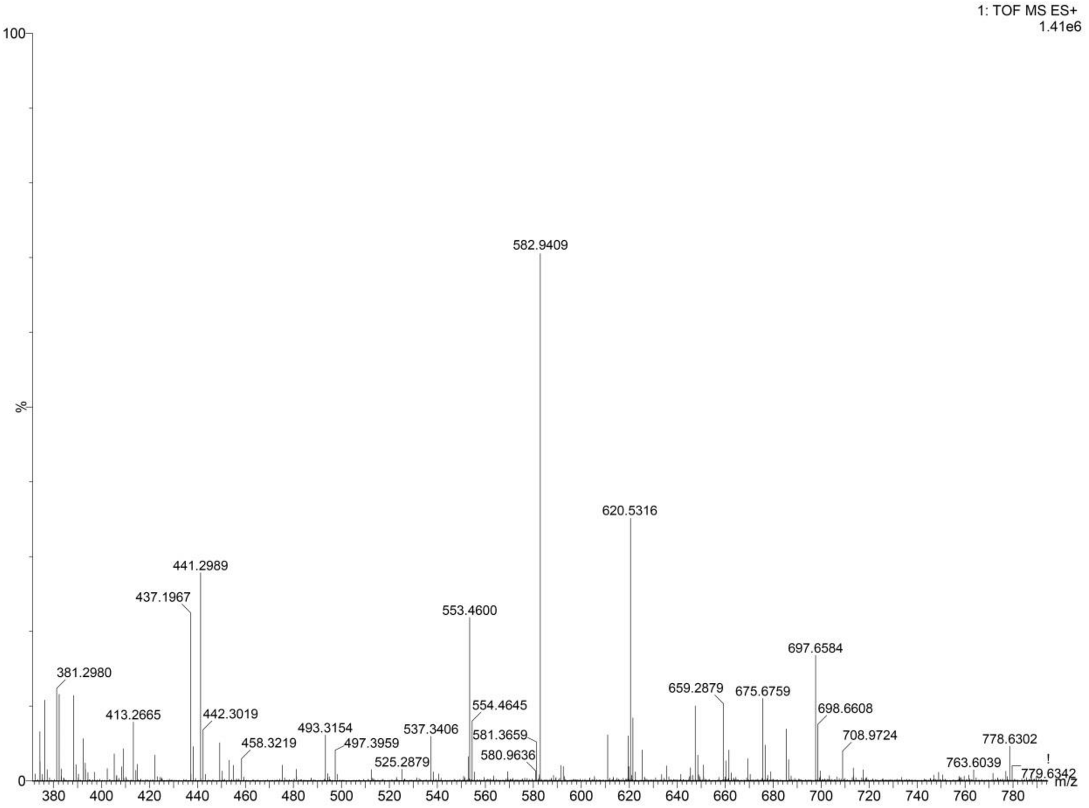

Figure S24. TOF MS of compound 22b.

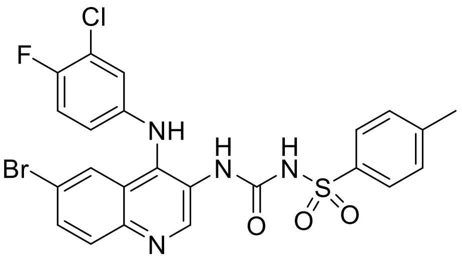

Compound 22c

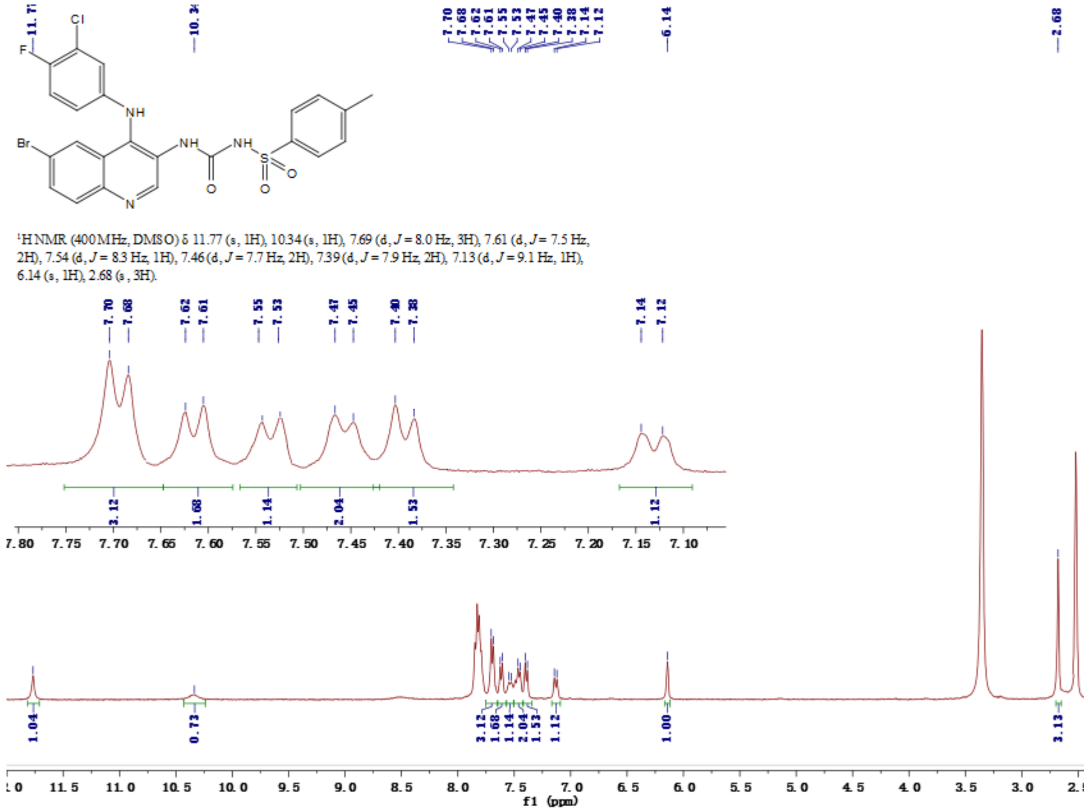

Figure S25. ¹H-NMR of compound 22c.

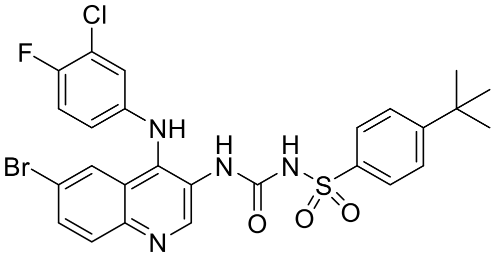

Compound 22d

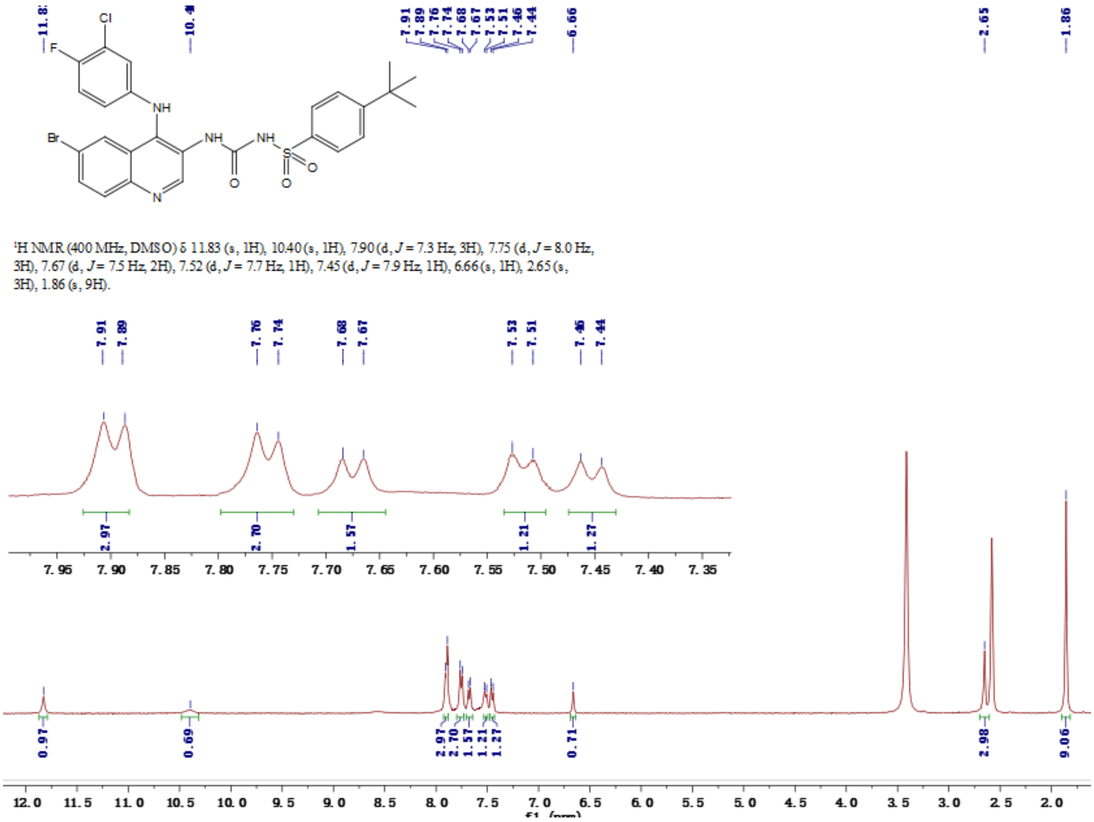

Figure S26. ^{1}H-NMR of compound 22d.

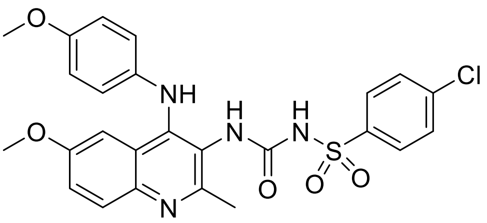

Compound 23a

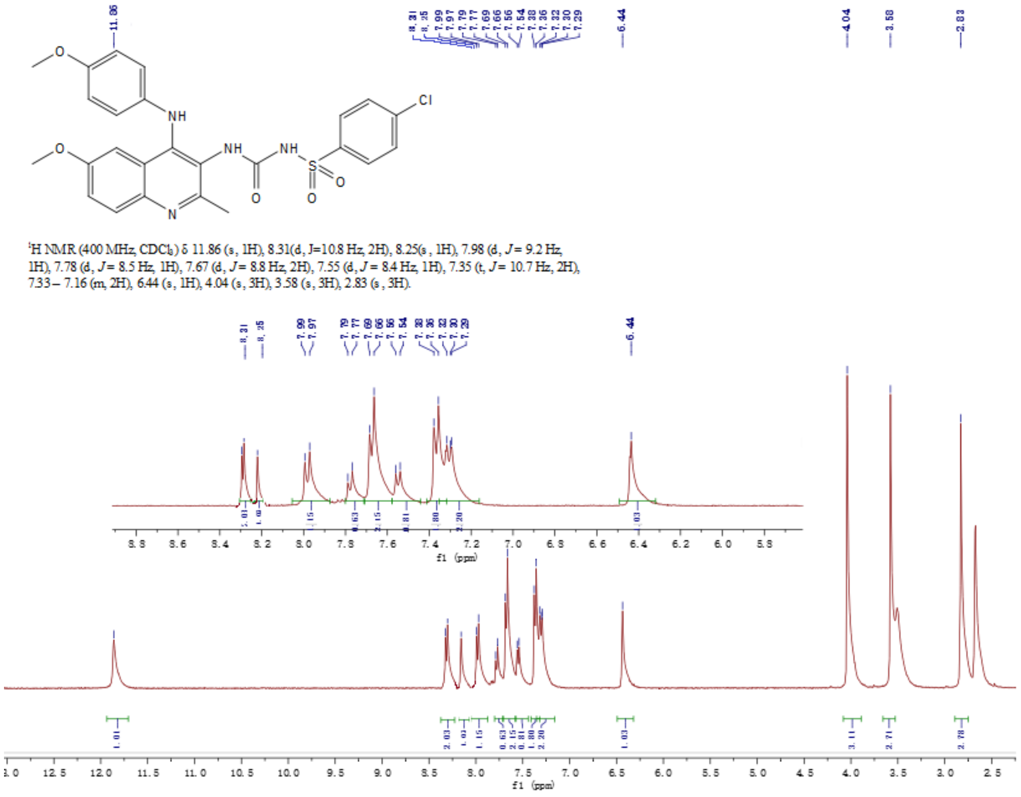

Figure S27. ^{1}H-NMR of compound 23a.

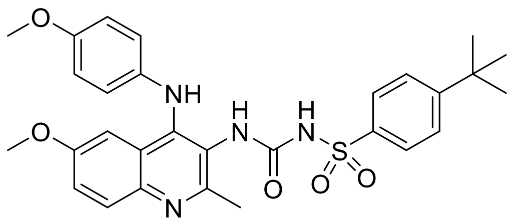

Compound 23b

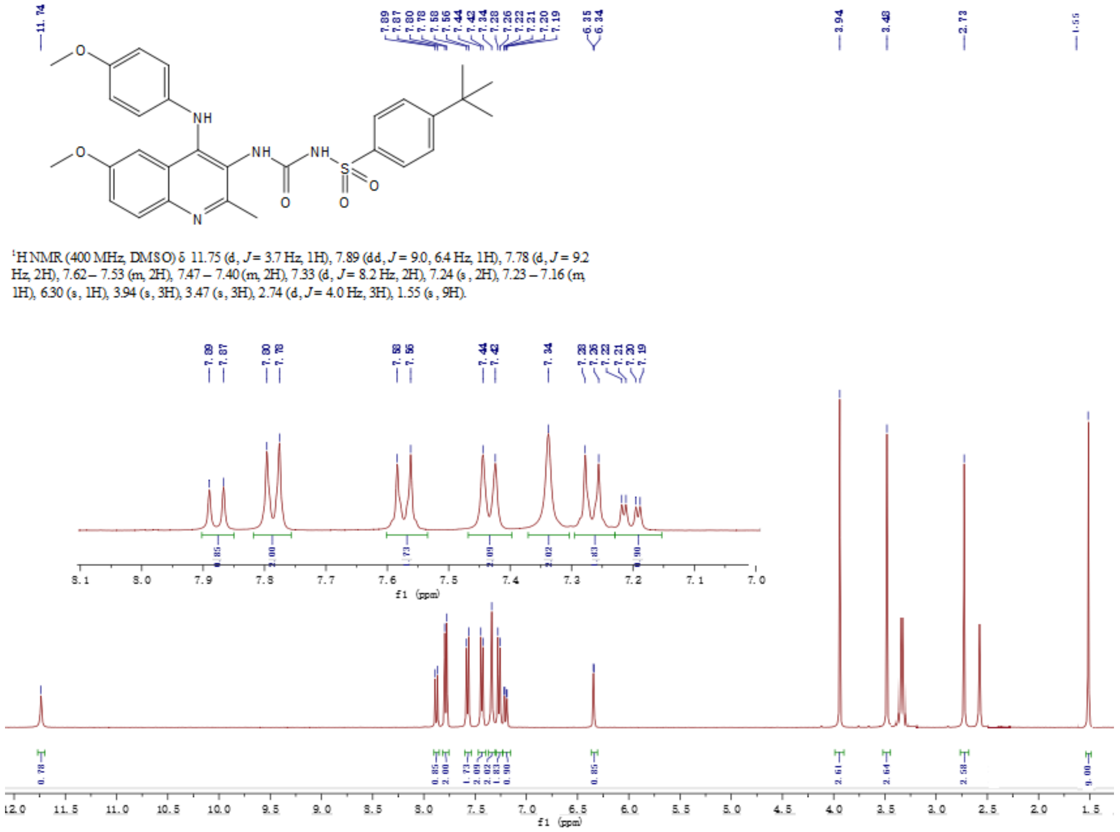

Figure S28. ^{1}H-NMR of compound 23b.

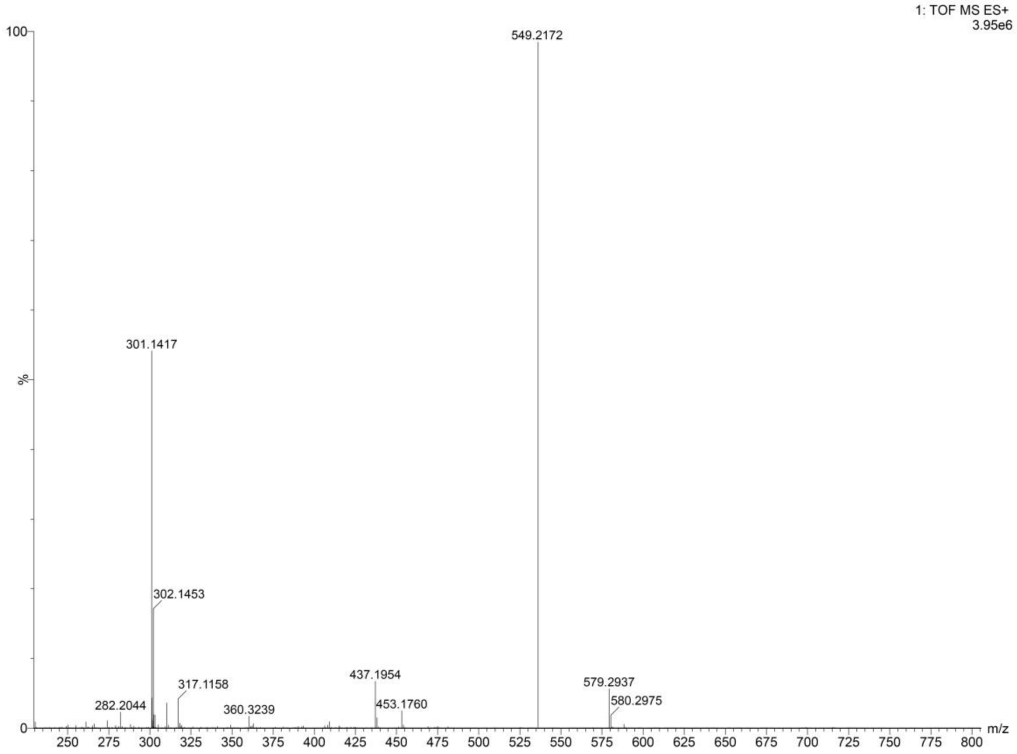

Figure S29. TOF MS of compound 23b.

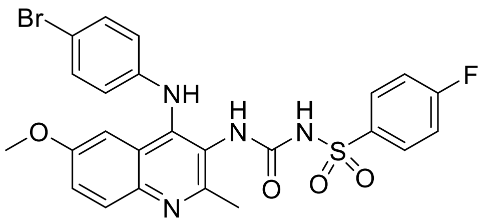

Compound 23c

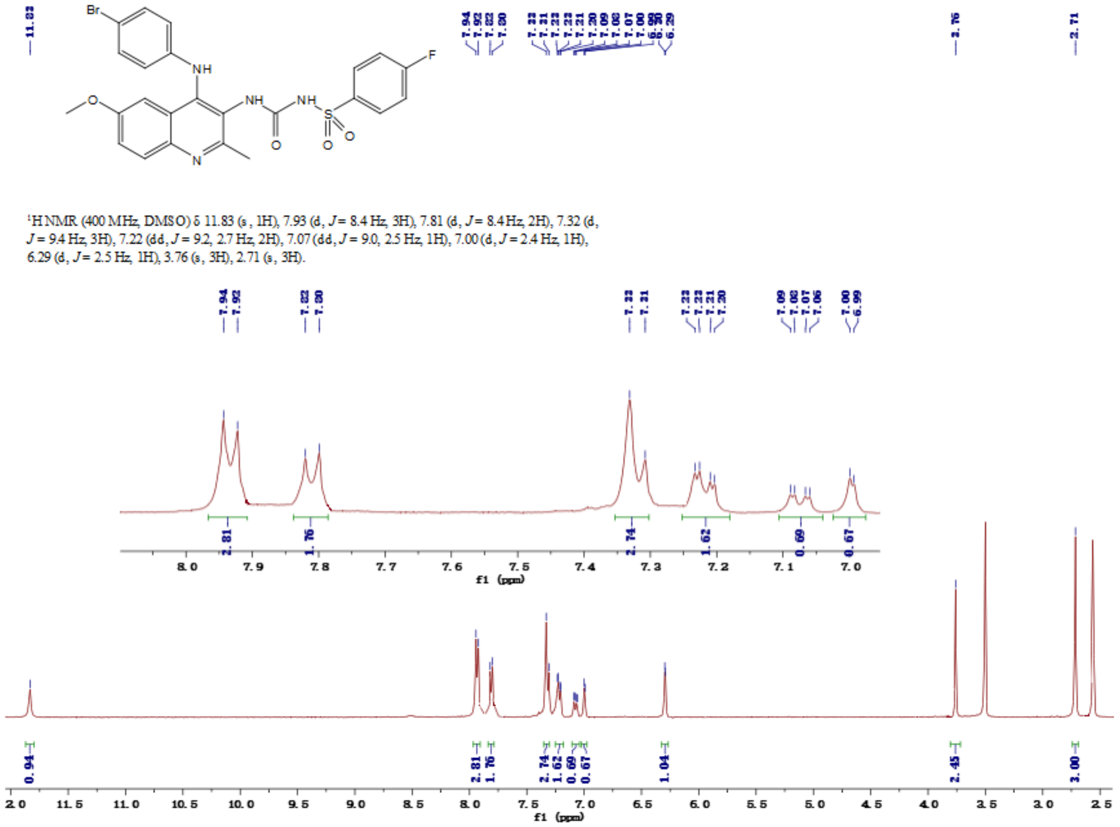

Figure S30. ^{1}H-NMR of compound 23c.

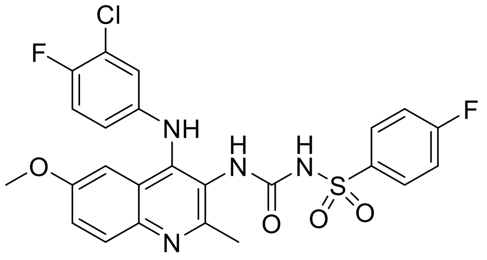

Compound 23d

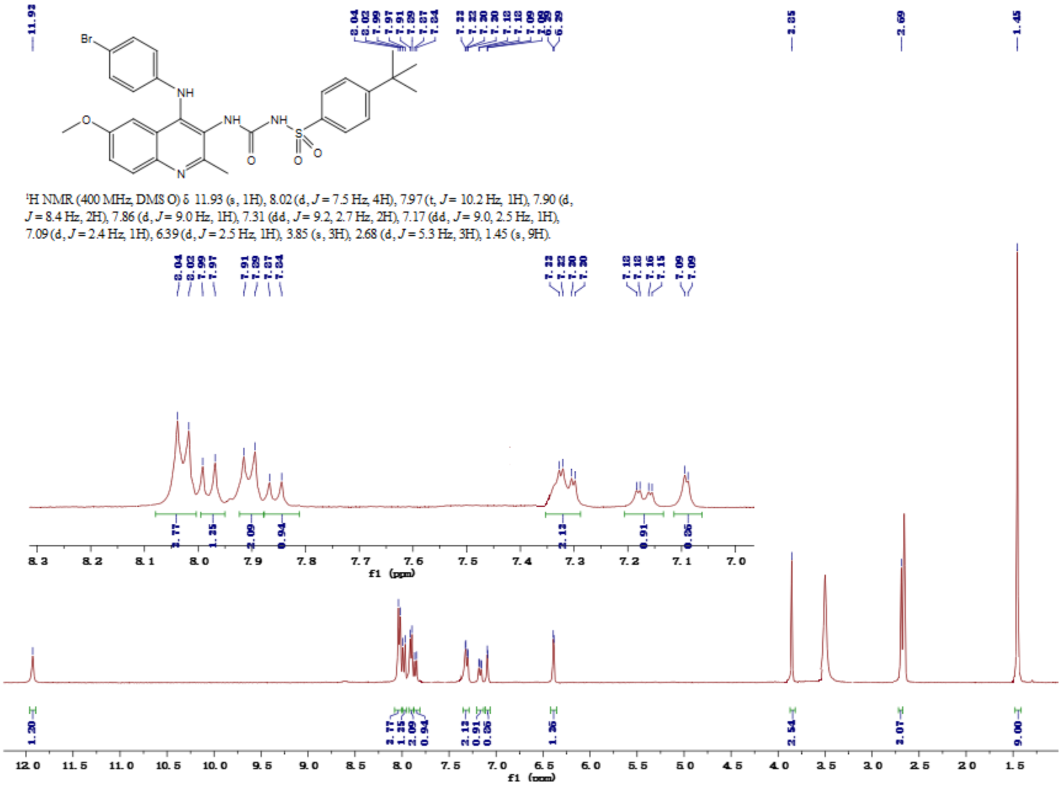

Figure S31. ^{1}H-NMR of compound 23d.
